# Origins and Evolution of Seasonal Human Coronaviruses

**DOI:** 10.3390/v14071551

**Published:** 2022-07-15

**Authors:** James R. Otieno, Joshua L. Cherry, David J. Spiro, Martha I. Nelson, Nídia S. Trovão

**Affiliations:** 1 Division of International Epidemiology and Population Studies, Fogarty International Center, National Institutes of Health, Bethesda, MD 20892, USA; jcherry@ncbi.nlm.nih.gov (J.L.C.); david.spiro@nih.gov (D.J.S.); nelsonma@mail.nih.gov (M.I.N.); 2 National Center for Biotechnology Information, National Library of Medicine, National Institutes of Health, Bethesda, MD 20894, USA

**Keywords:** seasonal coronaviruses, 229E, NL63, OC43, HKU1, evolution, zoonosis, recombination

## Abstract

Four seasonal human coronaviruses (sHCoVs) are endemic globally (229E, NL63, OC43, and HKU1), accounting for 5–30% of human respiratory infections. However, the epidemiology and evolution of these CoVs remain understudied due to their association with mild symptomatology. Using a multigene and complete genome analysis approach, we find the evolutionary histories of sHCoVs to be highly complex, owing to frequent recombination of CoVs including within and between sHCoVs, and uncertain, due to the under sampling of non-human viruses. The recombination rate was highest for 229E and OC43 whereas substitutions per recombination event were highest in NL63 and HKU1. Depending on the gene studied, OC43 may have ungulate, canine, or rabbit CoV ancestors. 229E may have origins in a bat, camel, or an unsampled intermediate host. HKU1 had the earliest common ancestor (1809–1899) but fell into two distinct clades (genotypes A and B), possibly representing two independent transmission events from murine-origin CoVs that appear to be a single introduction due to large gaps in the sampling of CoVs in animals. In fact, genotype B was genetically more diverse than all the other sHCoVs. Finally, we found shared amino acid substitutions in multiple proteins along the non-human to sHCoV host-jump branches. The complex evolution of CoVs and their frequent host switches could benefit from continued surveillance of CoVs across non-human hosts.

## 1. Introduction

There are four seasonal human coronaviruses (sHCoVs) that are endemic globally (229E, NL63, OC43, and HKU1), accounting for 5–30% of human respiratory tract infections [1]. CoV infections primarily involve the upper respiratory tract and the gastrointestinal tract, mostly producing mild respiratory diseases, but may sometimes cause life-threatening bronchiolitis and pneumonia in infants, young children, elderly, and immunocompromised individuals [2,3,4]. While the sHCoVs are globally distributed with a general seasonality between December and April, the frequency of detection varies by location and time [5,6,7,8,9,10,11]. Differences in clinical presentation and frequencies of detection by age and patient groups have been observed between the sHCoV infections and might be immune-mediated [1,11,12,13]. Repeat infections with the same sHCoV species are common, with up to a 21% re-infection rate over a six-month household study period [14,15]. However, due to the historic association with mild symptomatology, there have been limited long-term epidemiological studies on sHCoVs compared to other seasonal respiratory viruses such as the respiratory syncytial virus (RSV) and influenza virus [3,12]. Furthermore, there are no approved antiviral agents or vaccines for sHCoVs, with treatment being largely supportive.

**Figure 1:**
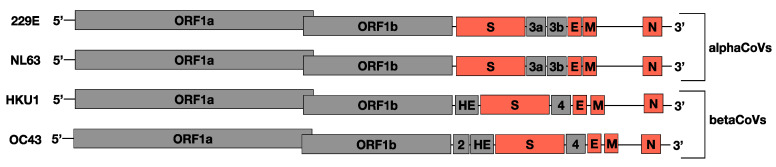
An illustration of the sHCoV genomes, not drawn to scale. The ORFs analyzed in this study are indicated in orange.

The four sHCoV species are part of two CoV genera, alphacoronavirus (alphaCoV, 229E, and NL63) and betacoronavirus (betaCoV, HKU1, and OC43) [16]. The sHCoVs are enveloped, non-segmented, positive-sense RNA viruses with genome sizes of 27–30 kb [17]. CoV genomes are quite diverse, in part due to a high frequency of RNA recombination [18,19]. About two-thirds of the genome comprises the open reading frame 1a/b (ORF1a/b) encoding two replicase polyproteins, with the remaining one-third consisting of ORFs encoding the structural proteins hemagglutinin-esterase (HE: 1.2–1.3 Kbp, in HKU1 and OC43 only), spike (S: 3.5–4.7 Kbp), envelope (E: 0.2 Kbp), membrane (M: 0.6–0.7 Kbp), and nucleocapsid (N: 1.1–1.3 Kbp), as well as several accessory proteins (Figure 1) [20]. The spike protein responsible for the characteristic crown-like appearance of these viruses under electron microscopy has utility in receptor binding and viral entry and is also the most variable at the nucleotide and amino acid levels of the coronaviruses’ ORFs [21]. The envelope and membrane proteins have a role in the CoV assembly and determining the shape of the viral envelope while the nucleocapsid protein has a protective role through the packaging of the viral genomic RNA into ribonucleoprotein complexes called nucleocapsids, ensuring timely replication and reliable transmission [22]. These four structural proteins are crucial for virus entry into host cells and subsequent replication.

Coronaviruses predominantly circulate among non-human hosts, with rare cross-species transmission to humans through handling of infected wild and domestic animals [23,24,25]. Until 2003, and prior to the identification of the severe acute respiratory syndrome coronavirus 1 (SARS-CoV-1) [26,27] as the causative agent of the SARS pandemic of 2002–2003 [28], only 229E and OC43, isolated nearly 60 years ago [29,30,31], were known to infect humans. Not long after the identification of SARS-CoV-1, NL63 and HKU1 were identified in 2004 and 2005, respectively [32,33]. It is thought that all sHCoVs originate from bat CoVs, with some transmitted directly to humans (NL63) and others indirectly transmitted through intermediate hosts (229E, HKU1, and OC43) [34,35,36]. The presumptive ancestor of the OC43 is the bovine CoV, with which it has been estimated to share ~97% genome identity [37,38], while 229E is thought to have emerged from a camelid CoV and shares ~91–93% genome identity [39,40,41]. Lastly, HKU1 is thought to have emerged from a murine CoV [42]. Understanding the zoonotic origins of sHCoVs not only aids in directing surveillance efforts for the future potential emergence of CoVs but also highlights cultural practices at the human and non-human interface that can help in designing prevention and treatment strategies.

While there have been few epidemiological studies on sHCoVs, evolutionary studies on the sHCoVs are even more limited. As we ponder the future trajectory of the SARS-CoV-2 virus, the endemic sHCoVs provide a unique window into CoV emergence and evolutionary history in the human population. In this study, we explored the evolutionary history of the sHCoVs using a dataset of 855 sequences from GenBank and examined the timing of emergence, zoonotic origins, genetic diversity, recombination patterns and rates, and potential adaptive protein changes. We used a multigene analysis approach that provides insight into the evolutionary histories of the spike, nucleocapsid, membrane, and envelope ORFs of these CoVs. We find that the origins of the sHCoVs are highly complex and uncertain, with our evolutionary reconstructions suggesting that different ORFs in a single sHCoV might have distinct origins. The complex and uncertain evolutionary histories of sHCoVs may be in part due to recombination and under sampling, particularly of non-human hosts, and a fuller understanding will be achieved with more sequence data and re-analysis.

## 2. Materials and Methods

### 2.1. Datasets and Subsampling

Dataset D1. We retrieved all coronavirus sequences available in GenBank as of 6th June 2020; approximately 65,000 sequences comprising at least 27 human and non-human hosts. This dataset was cleaned to remove sequences obtained from laboratory hosts, duplicates of the same isolate, samples that had undergone more than 10 passages, contaminants (e.g., primers and vaccines), sequences shorter than 50% of the respective protein or full genome length, and sequences for which no collection date could be reliably obtained either through GenBank metadata or publications. The dataset was subsequently restricted to the alphaCoV and betaCoV genera (to which the four sHCoV species belong), and focused the analyses on the envelope, membrane, nucleocapsid, and spike ORFs and whole genome sequences (WGS) (*n* = 8032 sequences, Figure S1).

Dataset D2. There was an obvious sampling bias in the dataset D1 by host and time (Figure S1), and these biases are known to impact phylodynamic inferences [43,44]. To address this, Maximum Likelihood (ML) trees (see Section 2.2. Maximum Likelihood (ML) Trees and Temporal Signal) were generated with the D1 dataset for each structural protein and WGS, and then downsampled each non-human CoV to ~30–40 sequences per host-clade (since some CoVs from the same host were represented by multiple clades) using the phylogenetic diversity analyzer software (PDA) that selects a subset of sequences that comprises the maximal phylogenetic diversity [45]. The earliest collected CoV sequence(s) for each host-clade were always retained in the PDA subsampled datasets (*n* = 2382 sequences).

Dataset D3. For computational efficiency, dataset D2 was further downsampled to a clade of coronaviruses with bootstrap support > 0.7 and a reasonably sufficient number of non-human hosts (Figure S2). In accordance with previous reports and observed preliminary clustering patterns on ML trees in this analysis, the HKU1 sequences were partitioned into two genotypes, A and B [8,46] (*n* = 1729 sequences).

Dataset D4. To avoid the potential impact of the much larger number sHCoV sequences in comparison with their respective non-human CoVs ancestors, the 229E and NL63 sequences were downsampled to an equal number of sequences as those of the 229E-related camelid CoVs [39,40,41]. For betaCoVs, the sHCoVs OC43 and HKU1 sequences were downsampled to an equal number as those of murine CoVs that were inferred to be closest phylogenetically [42]. Similar to Dataset D2, PDA was used for the downsampling, and the earliest isolated sHCoV sequence(s) for each species were always included in the subsampled datasets (*n* = 855 sequences, File S1).

Multiple sequence alignments were built using MAFFT v7.475 [47] and thereafter edited and cleaned manually using AliView v1.26 [48].

### 2.2. Maximum Likelihood (ML) Trees and Temporal Signal

For each dataset described above, an ML tree was generated using IQ-TREE {conda versions 2.0.3–2.1.4} [49], allowing the software to determine the best nucleotide substitution model [50] and using ultrafast bootstrap [51] approximation to estimate branch support. In order to visually examine the degree of temporal signal or accumulation of divergence in the datasets over the sampling time interval, the exploratory linear regression approach implemented in TempEst v1.5.3 [52] was employed. The root-to-tip divergences as a function of sampling time according to a rooting that maximizes the Pearson product moment correlation coefficient were plotted in TempEst. Outlier sequences that were either not divergent enough or too divergent were removed from the datasets. The new datasets were used to generate new ML trees that were heuristically time-transformed using TempEst and subsequently used as starting trees to reduce the burn-in of the Markov Chain Monte Carlo (MCMC) phylodynamic analyses (see Section 2.5. Phylodynamics and Host-Jump Analysis).

### 2.3. Comparative Genomics

With dataset D4, and using MEGAX [53], we calculated the mean sequence divergence over all sequence pairs for each CoV species. We used the maximum composite likelihood method [54], assumed a heterogeneous substitution pattern among lineages, and modeled evolutionary rates among sites using the gamma distribution with four rate categories.

The non-human CoVs selected for this formed a clade with the sHCoVs and were either paraphyletic to the sHCoVs or a sister group with bootstrap support >0.7. However, when the non-human CoVs that shared an MRCA with sHCoVs, rather than the ones in the more distant past, were represented by a single sequence, the mean pairwise genetic distance could not be calculated on the single sequence.

### 2.4. Recombination Analysis

Coronaviruses are known to frequently recombine [42,55,56,57]. We used ClonalFrameML v1.12 and dataset D4 to estimate the ratio of the rate of recombination to the rate of point mutation (*ρ*/*θ*, also referred to as *R*/*theta* or *ε*), the ratio of effects of recombination to point mutation (*r*/*m*), and the number of substitutions per recombination event (*δν*). The *R*/*theta*, *r*/*m,* and *δν* values were estimated not only within the alphaCoVs and betaCoVs but also within sHCoV species. To perform comparisons of these estimates between sHCoV species, we used subsets of dataset D4 that (i) had one sHCoV species and all other non-human CoVs {to estimate inter-host recombination rates} and (ii) had one sHCoV species {to estimate intra-species recombination rates}. However, since ClonalFrameML was designed to be used on bacterial full genome datasets, this analysis was only performed on WGS (29–32 Kbp) and the spike protein (4.1–4.7 Kbp), and caution should be taken when interpreting these results.

Dataset D5. Recombination can distort phylogenetic tree inference and interpretation [58,59,60]. Using RDP4 [61], recombinant sequence regions in dataset D4 were iteratively removed until no more recombination signals were detected. The software’s default settings were used except for specifying that the sequences were linear and adding the 3SEQ method to the five default methods (RDP, GENECONV, MaxChi, Bootscan, and SisScan). Recombination signals were considered present if they were detected by three or more methods. Sequences that lost more than 50% of the respective ORF or WGS lengths as a result of the removal of the recombinant regions were discarded (*n* = 783 sequences).

Dataset D6. To test for recombination between sHCoVs from different genera, all the sequences from dataset D4 for WGS and each ORF were combined into single datasets and the non-sHCoV sequences were discarded. Recombination analysis was performed on dataset D6 with RDP as described above (*n* = 346 sequences).

### 2.5. Phylodynamics and Host-Jump Analysis

We used the BEAST v1.10.4 package [62,63] and dataset D5 to estimate the timing of the emergence of each sHCoV species, evolutionary rates, and zoonotic origins. Conditioning on the host of the taxon, the host transition history was modeled as a non-reversible continuous-time Markov chain (CTMC) process while reconstructing the unobserved hosts at the ancestral nodes in each tree of the posterior distribution [64]. For sequences with incomplete collection dates (without day or day and month), we incorporated uncertainty in the sampling date. An HKY+G+I nucleotide substitution model [65] was specified together with a Skyline coalescent tree prior [66] and an uncorrelated relaxed clock model [67]. The MCMC sampling in BEAST was undertaken for at least 700 million steps for each run, with each dataset undergoing at least three independent runs. We combined the log and tree files using LogCombiner until the effective sampling sizes (ESS) were >200 as assessed by Tracer v1.7.1 [68]. The posterior tree distributions were summarized and annotated with TreeAnnotator, after discarding at least 10% of the trees as chain burn-in. The summarized maximum clade credibility (MCC) trees were visualized using FigTree v1.4.4 and ggtree [69].

We also estimated the number of host transitions (Markov jumps) in order to have a complete summary of the host transmission processes [70]. (GitHub location for XML files: to be updated upon final submission).

### 2.6. Selection Analysis

Using the Datamonkey server (Available online: https://www.datamonkey.org), we tested for positive selection along the host-jump branches leading to the sHCoVs using two separate methods: (i) the adaptive Branch-Site Random Effects Likelihood (aBSREL) method [71] and (ii) the Branch-site Unrestricted Statistical Test for Episodic Diversification (BUSTED) method [72]. Both aBSREL and BUSTED estimate the ratio of nonsynonymous to synonymous substitutions (ω), determine the optimal number of ω rate classes, and estimate the proportion of sites belonging to each ω rate class. For this analysis, we used BEAST MCC trees with recombination-free tree topologies (from dataset D5) and sequence alignments from dataset D4 with recombinant regions intact so as not to miss potential positive selection signals from such regions.

### 2.7. Amino Acid Substitution Analysis

We used BEAST MCC trees (from dataset D5) and translated nucleotide sequence alignments from dataset D4 to perform amino acid (AA) ancestral reconstruction [73] of historical CoVs in order to elucidate the AA changes that might have characterized the inter-host transmission of non-human CoVs into humans, as well as the evolution and adaptation of the sHCoVs within the human host. The ancestrally reconstructed AA changes were also compared with the AA changes characterizing the SARS-CoV-2 Wuhan-Hu-1 or a variant of concern (VOC). The dataset D4 sequences were aligned against the Wuhan-Hu-1 SARS-CoV-2 reference genome (NC_045512.2), after which, the reference sequence was removed prior to the ancestral reconstruction and AA analysis. Where there was an AA insertion in the sHCoVs relative to the Wuhan-Hu-1 SARS-CoV-2 reference, we used the X.Y positional notation where X is the reference genome position and Y is the nth sHCoV AA insertion, i.e., the 2 in AA position 8.2 indicates the second AA insertion at position 8 relative to the Wuhan-Hu-1 SARS-CoV-2 reference genome.

## 3. Results

### 3.1. Zoonotic Origins of the Seasonal Human Coronaviruses

To investigate the zoonotic origins of the four sHCoV species, we generated MCC trees from the WGS, and the spike, envelope, membrane, and nucleocapsid ORFs.

**Figure 2:**
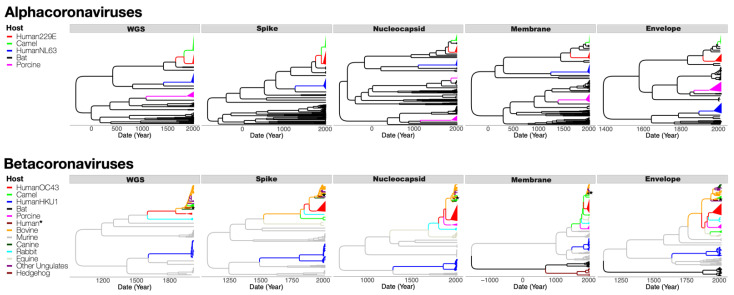
Maximum clade credibility (MCC) trees inferred from dataset D5 for full genomes (WGS), and the spike, nucleocapsid, membrane, and envelope ORFs, with the branches color-coded by the inferred coronavirus host. The upper panel shows MCC trees from alphacoronaviruses while the lower panel shows MCC trees from betacoronaviruses. Human, camel, and porcine coronavirus clades have been collapsed to increase readability. Human * is a lone human CoV (FJ415324) that clusters with ungulate and canine CoVs. Individual and more detailed MCC trees can be found in File S2.

For 229E, MCC trees generated from the WGS and the four ORFs had different topologies arising from recombination events deep in the viral evolutionary history. Bats are the ancestral host on each tree, but the phylogenetic relationships between the sHCoV 229E and clades associated with other CoV host species differ (Figure 2). The 229E and camelid CoVs formed a clade with very high branch support (posterior probability [PP] = 1) on the spike protein and WGS MCC trees. However, 229E was more closely related to bat CoVs than to camelid CoVs on trees inferred for the nucleocapsid, membrane, and envelope proteins. Analysis of the CoVs inter-host transmission process (Markov jumps {MJs}) for the envelope, membrane, and nucleocapsid estimated a 98–99% probability that the sHCoV 229E arose directly from a bat CoV (Table 1). For the spike protein and WGS, there was a 73% and 65% probability that 229E arose directly from a bat CoV, and a 27% and 34% probability that 229E originated from a camelid CoV, respectively. As camelid CoVs did not seem to have a higher probability of being ancestral to sHCoV 229E, we looked at the estimated origins of these camelid CoVs. It was estimated for the spike and WGS that there was a 61% and 35% probability, respectively, that the camelid CoVs arose from the sHCoV 229E. Taken together, there is a high degree of uncertainty as to whether camelids were intermediate hosts for the transmission of a bat CoV into humans or whether transmission occurred directly from a bat or potentially via an unsampled intermediate host, given the long branch lengths. This analysis also considers the possibility of a reverse zoonosis of the human 229E CoVs into camelids.

The zoonotic origins of the sHCoVs NL63 and HKU1 were inferred to be the bat and murine CoVs, respectively, with no apparent intermediate hosts (Figure 2 and Table 1). The two HKU1 A and B genotypes formed distinct and well-supported clades, with very long branch lengths, in all the MCC trees derived from the four ORFs (Figure 2).

Our analysis revealed that the sHCoV OC43 was a sister clade to a large group of CoVs from ungulate hosts (including bovine, buffalo, camel, deer, waterbuck, and yak) and canines, on MCC trees derived from WGS or the spike, nucleocapsid, and membrane proteins (Figure 2). Bovine CoVs were estimated to be the ancestor to OC43 with probabilities of 36%, 35%, 23%, 2%, and 20% for the WGS, spike, nucleocapsid, membrane, and envelope proteins, respectively (Table 1). The host with the highest probability of CoV origins for OC43 was a porcine CoV (25%) for the nucleocapsid, a camel CoV (75%) for the membrane, and a murine CoV (30%) for the envelope. While this analysis is consistent with a bovine CoV being the origin of the OC43 [37,38], it broadens the range of plausible origins to include murine, rabbit, canine, or other ungulate CoVs.

### 3.2. Rates of Evolution and Emergence Dates

**Figure 3:**
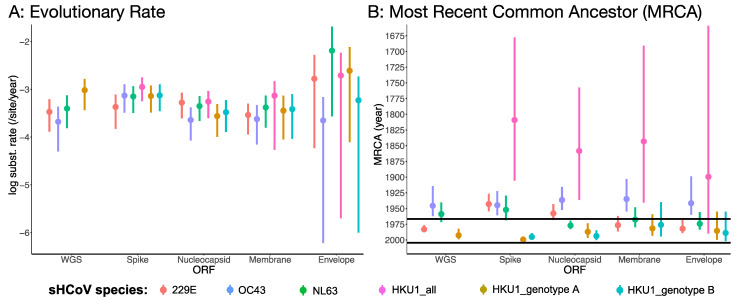
Estimates of the evolutionary rate (**A**) and MRCA age (**B**) for full genomes and four open reading frames (dataset D5) of the seasonal human coronavirus species. The black horizontal lines in (**B**) are the dates of first isolation for the 229E (1966), OC43 (1967), NL63 (2004), and HKU1 (2005). The WGS is missing data points for HKU1_all (collective for both genotypes) and HKU1_genotype B as sequences for HKU1_genotype B were all removed in the generation of recombination-free WGS dataset D5.

The mean substitution rates for the four sHCoVs were similar for the WGS and the four ORFs, ranging between 2.1 × 10^−4^ and 6.4 × 10^−3^ nucleotide substitutions/site/year and with overlapping 95% highest posterior density (HPD) (Figure 3A). However, the mean substitution rates for the ORFs had wider 95% HPDs than the full genomes, which is consistent with what would be expected from whole genomes that have more genetically informative sites in the estimation of the substitution rates. Nonetheless, even with the overlapping 95% HPD, HKU1 and OC43 coronaviruses often had the highest and lowest, respectively, mean substitution rates.

While the dates on which the sHCoVs were first isolated are well known [29,31,33,74], we were interested in estimating their MRCAs (Figure 3B and Figure S3). The median MRCA of the HKU1 viruses (HKU1_all) for the WGS, spike, nucleocapsid, and envelope was dated between 1809 and 1899 with wide 95% HPDs and was much older than the median MRCAs of the other sHCoVs (median: 1934–1983). However, HKU1 genotypes A and B had their median MRCAs dated much more recently, between 1975 and 1999, with overlapping 95% HPDs and we could not determine which of the two HKU1 genotypes might have emerged first. It is only for the spike ORF that the median MRCAs for the 229E, OC43, and NL63 appeared to follow the sequence of their respective first isolation dates. While the spike had the earliest median age of the MRCAs for 229E, NL63, and HKU1, this ORF had the youngest median MRCA ages for OC43 and HKU1 genotypes A and B. The estimated much earlier dating of HKU1 was not expected given these CoVs are the most recently isolated of the sHCoVs [33] and raises questions about whether this could be a consequence of under sampling and/or dating of two very distinct variants (genotypes A and B) that were independently transmitted into the human host.

### 3.3. Recombination Patterns

**Figure 4:**
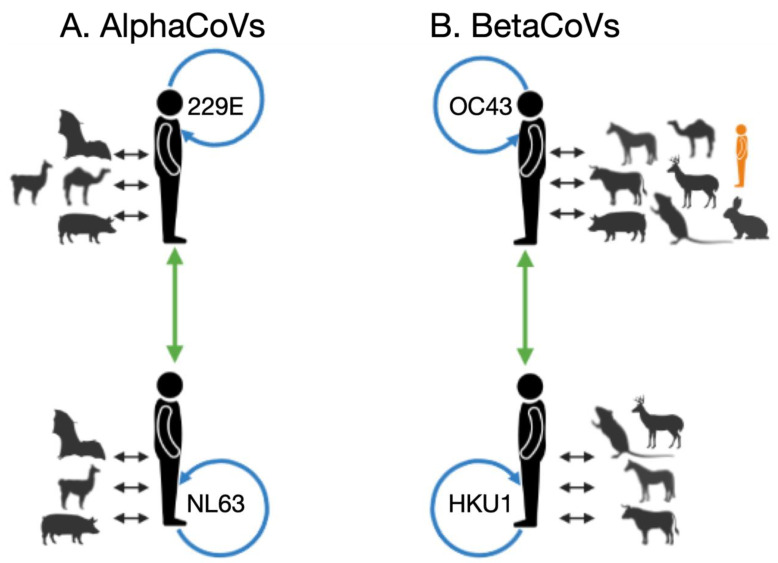
Summarized within and between host/species recombination patterns identified by RDP4, for alphaCoVs (**A**) and betaCoVs (**B**). For each sHCoV species, recombining CoVs are shown; non-human and sHCoV (black arrows), within sHCoV species (blue arrows), and between sHCoV species (green arrows). In orange is a lone human CoV (FJ415324) that clusters with ungulate and canine CoVs. Figure generated using Biorender.

Recombination is a known phenomenon in CoVs, and we identified recombination signals along the evolutionary history of the alpha and beta CoVs in our datasets (Figure 4 and File S3). With the exception of the envelope protein, recombination was detected by RDP4 in the membrane, nucleocapsid, spike, and WGS datasets. Some sHCoV sequences were characterized to be recombinants of CoVs from two or more hosts, as has similarly been shown for the SARS-CoV-2 spike protein [74]. Further inspection of the recombinant sequence pairs revealed that in addition to inter-host CoV recombination, there is recombination between human CoVs, both within and between sHCoV species (Figure 4). While intra-species recombination has previously been reported for genotypes within OC43 and HKU1 [46,75], and between a few NL63 isolates [76], we identified recombination within 229E as well as between the sHCoV species. However, recombination between sHCoV species was only observed between species in the same genus, i.e., between 229E and NL63 and between HKU1 and OC43, and was only identified in the spike and WGS datasets. Because sHCoV co-infections are frequently identified in the community and patient isolates [3,10], recombination within and between sHCoV species might be a frequent occurrence and could increase the breadth of the sHCoV species’ variants.

### 3.4. Recombination Rates

The observed frequency of recombination reflects both the rate of recombination events and the effects of selection. We assessed the apparent rates of recombination among CoVs and across the genome using ClonalFrameML which was designed to be used on bacterial genomes, therefore, caution should be taken when interpreting these results. We estimated that recombination happened up to 2 times more often than nucleotide changes due to point mutations (*R*/*theta*) in the spike protein, but 40 (betaCoVs) to 71 times (alphaCoVs) less often across the whole genome (Table 2a,b), indicating that the rate of recombination and/or patterns of selection might vary substantially along the genome with hotspots in certain genomic regions. The mean length of DNA imported by homologous recombination (*δ*) ranged from 209 bp (betaCoVs) to 336 bp (alphaCoVs) for the spike protein and 781 bp (betaCoVs) to 1033 bp (alphaCoVs) across the whole genome. Recombination overall was responsible for 29–42 times more substitutions than point mutation (*r*/*m*) in the spike, and approximately 0.8 times as many substitutions as point mutation across the whole genome. These estimates illuminate the substantial contribution of recombination in the evolution of CoVs, particularly in the spike protein.

With regard to differences in recombination rates between the alphaCoVs and betaCoVs genera (Table 2a,b), our estimates of *R*/*theta* were about two-fold higher in betaCoVs than alphaCoVs across the whole genome (0.025 vs 0.014) and within the spike protein (2.19 vs. 1.10). The estimated number of substitutions introduced by each recombination event (*δν*), however, was higher in the alphaCoVs than in the betaCoVs both for the spike (38 vs. 13 substitutions) and across the whole genome (59 vs. 36 substitutions). These results were consistent with subset datasets comprised of one sHCoV species and all other non-human CoVs (Table 2c). These observations imply that although the rate of recombination to point mutation is higher for the betaCoVs, more substitutions are introduced per recombination event for the alphaCoVs.

Nevertheless, the distinctive patterns of *R*/*theta* for alphaCoVs vs. betaCoVs were not consistent for the within sHCoV species recombination rate analysis (Table 2d) and instead followed the following order: HKU1 < NL63 < OC43 < 229E. This trend was reversed when considering the number of substitutions introduced by each recombination event, i.e., highest in HKU1 (67 substitutions) and lowest in 229E (2 substitutions). With regard to the HKU1 genotypes, there was a marked difference in the estimates of *R*/*theta* (0.2 vs. 0.06) and *δν* (2 vs. 37 substitutions) for genotypes A and B, respectively. The intra-genotype *R*/*theta* value for HKU1 genotype A was higher than that for NL63 while its corresponding *δν* value was only higher than that for 229E, depicting interesting variation in these *R*/*theta* and *δν* values between the two HKU1 genotypes and in comparison with the existing sHCoV species. We surmise that while sHCoV interspecies recombination rates vary mainly with genus (highest for betaCoVs), intraspecies recombination rates seem to vary with the sHCoV species’ first isolation date (highest for 229E and OC43, which have the earliest first isolates). This may be an artifact of the fact that recombination events are not detectable if the parents have not diverged sufficiently.

### 3.5. Pairwise Diversity

The estimated mean pairwise genetic diversity for each sHCoV is presented in Table 3. Genome-wide, 229E was estimated to be the least genetically diverse of the sHCoVs with HKU1 being the most genetically diverse, i.e., 229E < OC43 < NL63 < HKU1. This order of genetic diversity was similar to the order from ClonalFrameML’s number of substitutions introduced by each recombination event (*δν*). The HKU1 genotype B was independently more genetically diverse than 229E, NL63, and HKU1, while HKU1 genotype A was the least diverse. Of the four ORFs analyzed, the spike ORF was the most genetically diverse for the sHCoVs as well as for the CoVs from non-human hosts (Table 3 and Table S1).

Bat and murine CoVs that were sister groups to the sHCoVs were at least 7 to 9 times more diverse than the sHCoVs from the whole genome estimates (Table S1). In fact, murine CoVs were 62 and 14 times more diverse than the HKU1 genotypes A and B, respectively. Bat CoVs were the most diverse of the non-human CoVs while the ungulates-canines CoVs that share an MRCA with OC43 were the least diverse. Lastly, camelid CoVs that share an MRCA with 229E were less diverse compared to the human 229E CoVs.

### 3.6. Selection Analysis

We hypothesized that there might be positive selection on the CoV host-jump branches into humans for the envelope, membrane, nucleocapsid, and spike proteins due to their role in virus entry into host cells and subsequent replication. BUSTED did not infer any of the branches leading to the sHCoVs to be under positive selection (Table S2). On the other hand, aBSREL inferred the branches leading to the emergence of NL63, HKU1_all, and HKU1 genotype B to be under positive selection for the nucleocapsid protein. However, the high ω values estimated for a minority of codons seem unrealistically high, and likely reflect misalignment of a minority of codons rather than adaptation.

### 3.7. Amino Acid Substitutions

**Figure 5:**
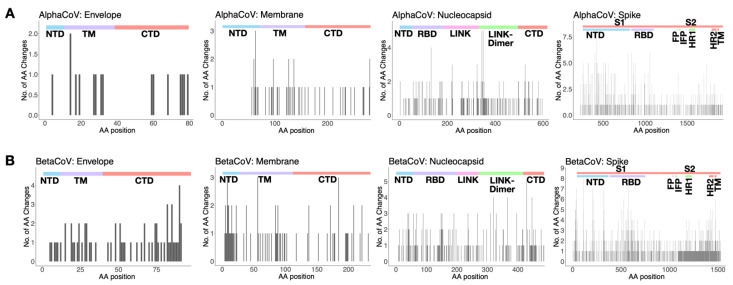
The number of inferred amino acid changes (AA) associated with the sHCoVs for AA positions in the envelope, membrane, nucleocapsid, and spike proteins from datasets D4 and D5. Panel (**A**) represents the aggregated AA changes from the alphaCoVs 229E and NL63 while (**B**) represents the aggregated changes from the betaCoVs OC43 and HKU1. At the top of each plot, the functional domains or regions of the respective proteins are shown; NTD = N-terminal domain, TM = transmembrane domain, CTD = C-terminal domain, RBD = receptor binding domain, LINK = central linker domain, LINK-Dimer = dimerization domain, S1 subunit, S2 subunit, FP = fusion peptide, IFP = internal fusion peptide, HR1 = heptad repeat 1, and HR2 = heptad repeat.

Amino acid (AA) ancestral reconstruction revealed that 29–57%, 29–35%, 16–26%, and 19–48% of the AA alignment positions of the spike, nucleocapsid, membrane, and envelope, respectively, had at least one AA substitution across the genealogy of the sHCoVs (File S4). In Figure 5 (and Figure S4), we show the number of the inferred AA changes associated with the sHCoVs in the envelope, membrane, nucleocapsid, and spike proteins. While AA changes were inferred to occur throughout the spike protein, an elevated number of changes occurred within the receptor binding domains (RBDs).

The majority of the AA changes along the host-jump branches in humans were consistently associated with NL63 and HKU1 for all four proteins analyzed, individually accounting for 25–62% of the AA changes along these host-jump branches. However, for the within-human branches, HKU1 and OC43 were associated with the largest numbers of AA changes, with the exception of the spike protein where 229E had the second highest number of AA changes after HKU1. It is important to note that the sHCoV species associated with the most AA changes along the host-jump and within-human branches had the longest branch lengths and the largest synonymous changes of all the sHCoVs, and therefore, the higher number of AA changes does not necessarily reflect differences in selection.

For all four structural proteins, there was no single common AA position at which there were AA changes for all the four host-jump branches leading to the emergence of the sHCoV species. This might not be surprising considering that the four sHCoVs have different zoonotic origins (see Section on Zoonotic origins of the seasonal human coronaviruses). However, the following positions had AA changes in the emergence of three sHCoV species: spike [152, 624, 680, 932, 1153, 1154, 1165, 1170], nucleocapsid [127], membrane [222], and envelope [27] (Table 4). Most of the shared positions in the sHCoV host-jump branches were also observed to have AA changes in 1–15 non-human to non-human host-jump branches and/or within-host branches in the MCC trees.

While the majority of the AA changes at these common AA positions along the host-jump branches did not yield any obvious patterns, we broadly categorized some of the observed AA changes into (i) convergent changes to the same AA {e.g., NL63: L1224F and HKU1: G1224F}, (ii) divergent changes from the same AA to different AAs {e.g., NL63: I769L and 229E: I769N}, (iii) parallel AA changes {e.g., NL63: A1009S and 229E: A1009S}, (iv) convergent AA changes with SARS-CoV-2 {e.g., OC43: N856K (Omicron VOC)}, (v) completely reversed AA changes between sHCoV species {e.g., NL63: I1225V and HKU1: V1225I}, and (vi) partially reversed AA changes between sHCoV species {e.g., NL63 S162Y and HKU1: N162S} (Table S2).

The host-jump branches leading to sHCoVs NL63, HKU1, and OC43 experienced convergent AA changes with SARS-CoV-2 Wuhan-Hu-1 or a VOC in the spike protein, i.e., NL63: P80A {Beta VOC} [77], T375S {Wuhan-Hu-1} [78], HKU1: E339D {Omicron VOC} [79], F371L {Omicron VOC} [80], R969K {Omicron VOC}, and OC43: N856K {Omicron VOC} [81]. For the nucleocapsid and membrane proteins, HKU1 {E63D} and NL63 {L82I}, respectively, had convergent AA changes with Wuhan-Hu-1 CoV.

## 4. Discussion

Using a multigene and a complete genome approach, we find the evolutionary histories of sHCoVs to be highly complex, owing to frequent recombination of CoVs, including within and between sHCoVs, which occurs at different rates. The origins of sHCoVs 229E and OC43 may depend on the gene studied. For HKU1, we propose the possibility of two independent non-human to human transmission events. We find shared AA substitutions along the non-human to sHCoV host-jump branches, even though these branches did not often appear to be under positive selection. However, these observations are limited by a patchy sampling of animal viruses. Tracking the evolution of the sHCoVs will not only aid the understanding of their evolutionary history and the mechanisms for the potential emergence of novel human CoVs but also has implications for the development of diagnostics, vaccines, and therapeutics.

The emergence of 229E from a bat CoV has been thought to be through camelids as intermediate hosts based on phylogenetic clustering, genetic similarity to, and serological detection of similar viruses in dromedary camels and alpacas [39,40,41]. In our analysis, the camelid CoV origins for 229E were not well supported, and instead, independent transmissions of bat CoVs into humans and camelids appeared more likely. However, the possibility of reverse zoonosis of 229E into camelids would need further scrutiny as this would be in contrast with the MERS CoV whereby it is thought that humans represent an evolutionary dead-end for MERS-CoV [82].

For OC43, a less certain origin was observed than the previously proposed emergence from a bovine CoV; sHCoV OC43 clustered with ungulate and canine CoVs whose sequence data was not available at the time the bovine origin hypothesis was put forward [37]. We estimated bovine CoV to have a 2–36% probability as the origin of the OC43, and regarding some of the ORFs, porcine, camel and murine CoVs had the highest probabilities for the origins of OC43. Our observations widen the repertoire of potential OC43 origin/intermediate hosts to include other ungulates, rabbits, and canines; hosts that are either domesticated or wild but always in close proximity to humans.

Coronaviruses frequently recombine [18], and SARS-CoV-2 is already showing signs of recombination [83]. The process of recombination confers increased viral diversity and potential for inter-host transmission [56,84]. We observed the sHCoVs to be recombinants of non-human CoVs, indicating that either the sHCoVs that emerged in humans were already recombinants of diverse non-human CoVs or the extant sHCoVs are a product of continuous viral back-and-forth transmission between human and non-human hosts. We report previously undocumented recombination within 229E, as well as recombination between the sHCoV species from the same genera. Our results demonstrate that recombination frequently occurs beyond the spike protein which is often the focus of recombination analyses. As viral co-infections are frequently identified in patients [3,10], recombination between sHCoV species could be a real-world phenomenon that needs to be considered in studying the evolution of human CoVs in the SARS-CoV-2 era. To what extent do these within and between sHCoV species recombination events facilitate the expansion of sHCoV variants and, thereby, the fitness of these sHCoVs warrants further investigation. Moreover, our observations raise questions about recombinant variants that might be missed by current sHCoV diagnostic panels.

Replication-associated mutations are a major source of genetic diversity in RNA viruses [85]. Recombination rates varied across the genome, between alpha and beta CoV genera, and between the sHCoVs. The higher rates of recombination in the spike protein relative to other parts of the genome have also been observed in other studies using different methods [56]. Two recent papers looking at rates of adaptation [86] and recombination [87] in sHCoVs reported that these two rates are concurrently elevated and decreased in different parts of the genome. The two studies corroborate our observations on recombination rate variation across the sHCoV genomes and highlight the important role of recombination in these CoVs’ adaptive processes, considering that recombination was estimated to cause 0.8 times as many substitutions as point mutation events across the whole genome.

It was surprising that the MRCA of the two HKU1 genotypes analyzed collectively (HKU1_all) was dated much earlier than all the other sHCoVs, considering that HKU1 is the most recently isolated sHCoV [33]. A previous analysis using only the spike protein S1 domain of HKU1 had estimated MRCA dates in the 1950s [88]. Since the MRCAs of HKU1 A and B genotypes are herein dated more recently and a few years prior to the HKU1 first isolation date (similar to the lapse between the estimated MRCA and initial isolation dates for 229E and OC43), we propose two scenarios: (i) a single inter-host transmission into the human population with prolonged undetected circulation or (ii) two independent inter-host transmission events into the human population of two closely related but very distinct viruses at similar time periods. Insufficient sampling is often characterized by large uncertainty (95% HPDs) in MRCA estimates [89] as was observed with HKU1. In addition, retrospective sampling has led to the identification of NL63 isolates such as the KF530110 (NL63/human/USA/838-9/1983) that are older than the first ascribed NL63 isolate from 2005, and therefore, it is not farfetched to think that HKU1 might have been in circulation in the human population without prior isolation and identification. Two independent transmission events at similar time periods could be supported by the consistently phylogenetically distinct HKU1 genotypes in multiple ORFs, in addition to the marked variation in genetic diversity and recombination rates. However, the best evidence for the independent transmission of each HKU1 genotype into the human host would be the phylogenetic clustering of each genotype with a murine CoV as a sister clade. Additionally, it would be worthwhile to investigate if the two HKU1 genotypes are characterized by distinct viral phenotypes such as clinical presentation and frequency of detection.

It is thought that the criteria that determine a virus’s inter-host transmission are the availability of host receptor for virus binding and entry, the permissiveness of the host cells to allow the virus to replicate, and avoidance of the host species-specific innate immune responses inhibiting viral replication [90]. Therefore, a virus that has undergone a cross-species transmission will leave some adaptive footprint in its amino acid profile. Ancestral reconstruction of the sHCoV spike, nucleocapsid, membrane, and envelope protein sequences revealed that at least 16% and up to 57% of the AA positions in these proteins had at least one AA substitution across the genealogy of the sHCoVs. Of the four structural proteins, the spike protein had the largest number of one amino acid substitutions, which is expected because it is the longest of these four proteins and also has a more significant role in the adaptation of CoVs to new hosts [56]. However, except for the host-jump branches leading to NL63, HKU1_all, and HKU1 genotype B for the nucleocapsid, the inter-host transmission process leading to the emergence of the sHCoVs did not appear to be under positive selection. Noteworthily, these host-jump branches may not be under adaptation as HKU1 and NL63 had the longest host-jump and within-host branches, largest synonymous changes, and in some cases had unrealistically high ω values. A recent computational study of the spike protein inferred adaptive evolution in 229E and OC43 viruses and none at all in HKU1 and NL63 [86]. Taken together, it could be that the inter-host transmission of CoVs from a non-human to a human host, as well as continued circulation in the human population, can occur even without detectable positive selective pressure that is often expected to drive the optimization of a virus to its host [91].

### Study Limitations

There is a very limited sampling of animal viruses. While our observations improve on the current understanding of the zoonotic origins and evolution of the sHCoVs, these inferences are limited by the sparse sampling of the animal viruses.

## 5. Conclusions

As a consequence of the CoVs’ ability to recombine, mutate, and infect multiple hosts, SARS-CoV-2 is certainly neither the last CoV to spill over into the human population nor the last with pandemic potential. As more human and non-human viral genome sequences become available, there is an opportunity and necessity to further investigate the evolutionary histories of CoVs to achieve a better understanding of how these viruses jump between species and establish recurrent infections. These investigations could lead to the design of appropriate control strategies. Continuous surveillance of non-human CoV hosts would be essential for the early identification of potential zoonotic outbreaks, especially in largely under-sampled areas such as Africa whose bat CoVs appeared ancestral to the sHCoVs 229E and NL63 [40,41].

## Figures and Tables

**Table 1:** The estimated zoonotic origins of the four species of human seasonal coronaviruses inferred from BEAST Markov Jumps (MJs) for whole genomes and the envelope, membrane, nucleocapsid, and spike ORFs. For 229E and OC43, we included the percentage of MJs of the presumptive ancestors, other potential ancestors with a percentage higher than the presumptive ancestor, or CoVs whose cumulative percentage of MJs were ≥70%.

Genus	Species	WGS/ORF	Zoonotic Origins of the Human Coronavirus {% Markov Jumps}
alphaCoVs	229E	WGS	Bat {65} Camelid {34}
		Spike	Bat {73} Camelid {27}
		Nucleocapsid	Bat {99}
		Membrane	Bat {98}
		Envelope	Bat {99}
	NL63	WGS	Bat {97}
		Spike	Bat {99}
		Nucleocapsid	Bat {95}
		Membrane	Bat {97}
		Envelope	Bat {98}
betaCoVs	HKU1	WGS	Murine {83}
		Spike	Murine {92}
		Nucleocapsid	Murine {72}
		Membrane	Murine {78}
		Envelope	Murine {71}
	OC43	WGS	Bovine {36} Murine {34} Rabbit {10} Canine {9}
		Spike	Bovine {35} Murine {19} Camel {14} Rabbit {14} Canine {8} Equine {5}
		Nucleocapsid	Porcine {25} Bovine {23} Murine {18} Rabbit {15} Equine {10}
		Membrane	Camel {75} Murine {6} Rabbit {3} Porcine {3} Equine {2} Bovine {2} Bat {2}
		Envelope	Murine {30} Porcine {24} Bovine {20} Equine {8} Rabbit {6} Camel {3}

**Table 2:** Estimates of the ratio of recombination rate to point mutation rate (*R*/*theta*), substitutions by recombination relative to point mutation (*r*/*m*), number of substitutions per recombination event (*δν*), and mean length of DNA imported by homologous recombination (*δ*) from dataset D4. For each genome segment and analysis, the highest values are shown in bold. These analyses were conducted for the two HKU1 genotypes collectively (HKU1_all) and independently.

Genome Segment	Coronavirus Genus/Species	*R*/*Theta*	*r*/*m*	*δν*	*δ*
(a) Spike	alphaCoVs	1.103	**41.590**	**37.718**	**335.413**
	betaCoVs	**2.193**	28.494	12.994	208.896
(b) WGS	alphaCoVs	0.014	0.840	**58.043**	**1032.937**
	betaCoVs	**0.025**	**0.867**	35.369	780.250
(c) WGS: Interspecies analysis (between one sHCoV species and all other non-human CoVs)	229E	0.013	0.741	**57.465**	**1146.815**
	NL63	0.016	**0.943**	**59.561**	**970.252**
	OC43	**0.027**	0.888	32.291	687.266
	HKU1_all	**0.025**	**0.890**	35.890	760.242
	HKU1 Genotype A	0.024	0.840	35.170	747.680
	HKU1 Genotype B	0.024	0.865	36.718	787.036
(d) WGS: Intraspecies analysis (within one sHCoV species)	229E	**1.693**	**2.915**	1.722	649.287
	NL63	0.138	0.885	**6.415**	106.019
	OC43	**0.329**	1.636	4.967	**675.283**
	HKU1_all	0.029	**1.912**	**66.927**	**743.307**
	HKU1 Genotype A	0.216	0.537	2.493	176.197
	HKU1 Genotype B	0.056	2.123	37.595	811.754

**Table 3:** Mean pairwise genetic distances for each sHCoV species derived from WGS and four ORFs from dataset D4. In bold is the highest pairwise distance for each ORF/WGS and underlined is the highest pairwise distance for each sHCoV species.

sHCoV Species	WGS	Spike	Envelope	Membrane	Nucleocapsid
229E	0.0048	0.0348	0.0040	0.0079	0.0121
NL63	0.0081	0.0362	0.0077	0.0081	0.0076
OC43	0.0076	0.0268	0.0085	0.0082	0.0077
HKU1_all	**0.0222**	**0.1040**	**0.0795**	**0.0176**	**0.0231**
HKU1 Genotype A	0.0028	0.0049	0.0010	0.0020	0.0062
HKU1 Genotype B	0.0122	0.0120	0.0072	0.0039	0.0049

**Table 4:** A list of positions with AA change in at least two host-jump branches leading to the sHCoVs. Where there was an AA insertion in the sHCoVs relative to the Wuhan-Hu-1 SARS-CoV-2 reference genome, we used the X.Y positional notation where X is the reference genome position and Y is nth sHCoV AA insertion. The positions at which there were no AA changes in branches other than the host-jump branches leading to the sHCoVs are in bold.

ORF	Positions Shared by Alpha sHCoVs 229E and NL63	Positions Shared by Beta sHCoVs OC43 and HKU1	Positions Shared between Alpha and Beta sHCoVs
Spike	**769**, 1009, 1210, and 1253	8.21, 8.27, 8.31, 146, 154, 257, **329**, 463.11, 489, 490.5, **490.7**, 490.9, 497, 504.51, 1135, 1181, and 1221	60, 74, 152, 162, 208, **271**, 295, **321,** 367, 441, 476, 580, **624**, 628, 629, **641**, 680, 684, 751, 776, 795, 817, 932, 939, 957, 975, 1061, 1071, 1076, 1101, 1104, 1116, 1124, 1142, 1153, 1154, 1165, 1170, 1171, 1174, 1185, 1192, 1224, 1225, 1226, 1227, 1228, and 1272
Nucleocapsid	158, 160, 205, 216, 236, 252.7, 366, and 402	32 and 373.3	37, 62, 125, 127, 166, 182, 208, 210, 212, 240, 241, 249, 256, 297, 321, 323, 354, 364, 365, 384, 389, 390, 392, and 394
Membrane	8, 11, **72**, and **82**	-	10, **12**, 15, 188, and **222**
Envelope	-	-	3, 26, 27, 66, **72**, and 73

## Data Availability

No new data were generated or analyzed in support of this research. The XML-format files containing the data and model parametrization will be made available on GitHub (https://github.com/jrotieno/seasonal_HCoV_origins).

## Supplementary Materials

The following supporting information can be downloaded at: https://www.mdpi.com/article/10.3390/v14071551/s1, Figure S1: Downloaded and cleaned dataset D1; Figure S2: Maximum Likelihood phylogenetic trees from dataset D2; Figure S3: Estimated dates for the MRCAs and parent to the MRCAs; Figure S4: Number of inferred amino acid changes per sHCoV species per ORF; Table S1: Mean pairwise genetic distances; Table S2: Test for positive selection in the emergence of sHCoV species; Table S3: Select amino acid changes along the non-human to sHCoV host-jump branches. Supplementary File S1: subsampling_data.xlsx. Supplementary File S2: MCC Trees. Supplementary File S3: RDP Recombination Analysis. Supplementary File S4: Amino acid changes.

## References

[1] Su S., Wong G., Shi W., Liu J., Lai A.C.K., Zhou J., Liu W., Bi Y., Gao G.F. (2016). Epidemiology, Genetic Recombination, and Pathogenesis of Coronaviruses. Trends Microbiol..

[2] Kuypers J., Martin E.T., Heugel J., Wright N., Morrow R., Englund J.A. (2007). Clinical Disease in Children Associated With Newly Described Coronavirus Subtypes. Pediatrics.

[3] Otieno G.P., Murunga N., Agoti C.N., Gallagher K.E., Awori J.O., Nokes D.J. (2020). Surveillance of Endemic Human Coronaviruses (HCoV-NL63, OC43 and 229E) Associated with Childhood Pneumonia in Kilifi, Kenya. Wellcome Open Res..

[4] Falsey A.R., Walsh E.E., Hayden F.G. (2002). Rhinovirus and Coronavirus Infection—Associated Hospitalizations among Older Adults. J. Infect. Dis..

[5] Esper F., Weibel C., Ferguson D., Landry M.L., Kahn J.S. (2006). Coronavirus HKU1 Infection in the United States. Emerg. Infect. Dis..

[6] Koetz A., Nilsson P., Lindén M., van der Hoek L., Ripa T. (2006). Detection of Human Coronavirus NL63, Human Metapneumovirus and Respiratory Syncytial Virus in Children with Respiratory Tract Infections in South-West Sweden. Clin. Microbiol. Infect..

[7] Suzuki A., Okamoto M., Ohmi A., Watanabe O., Miyabayashi S., Nishimura H. (2005). Detection of Human Coronavirus-NL63 in Children in Japan. Pediatr. Infect. Dis. J..

[8] Lau S.K.P., Woo P.C.Y., Yip C.C.Y., Tse H., Tsoi H.-W., Cheng V.C.C., Lee P., Tang B.S.F., Cheung C.H.Y., Lee R.A. (2006). Coronavirus HKU1 and Other Coronavirus Infections in Hong Kong. J. Clin. Microbiol..

[9] Sipulwa L.A., Ongus J.R., Coldren R.L., Bulimo W.D. (2016). Molecular Characterization of Human Coronaviruses and Their Circulation Dynamics in Kenya, 2009–2012. Virol. J..

[10] Nyaguthii D.M., Otieno G.P., Kombe I.K., Koech D., Mutunga M., Medley G.F., Nokes D.J., Munywoki P.K. (2021). Infection Patterns of Endemic Human Coronaviruses in Rural Households in Coastal Kenya. Wellcome Open Res..

[11] Gaunt E.R., Hardie A., Claas E.C.J., Simmonds P., Templeton K.E. (2010). Epidemiology and Clinical Presentations of the Four Human Coronaviruses 229E, HKU1, NL63, and OC43 Detected over 3 Years Using a Novel Multiplex Real-Time PCR Method. J. Clin. Microbiol..

[12] Nickbakhsh S., Ho A., Marques D.F.P., McMenamin J., Gunson R.N., Murcia P.R. (2020). Epidemiology of Seasonal Coronaviruses: Establishing the Context for the Emergence of Coronavirus Disease 2019. J. Infect. Dis..

[13] Cabeça T.K., Granato C., Bellei N. (2013). Epidemiological and Clinical Features of Human Coronavirus Infections among Different Subsets of Patients. Influ. Other Respir. Viruses.

[14] Kiyuka P.K., Agoti C.N., Munywoki P.K., Njeru R., Bett A., Otieno J.R., Otieno G.P., Kamau E., Clark T.G., Van Der Hoek L. (2018). Human Coronavirus NL63 Molecular Epidemiology and Evolutionary Patterns in Rural Coastal Kenya. J. Infect. Dis..

[15] Munywoki P.K., Koech D.C., Agoti C.N., Lewa C., Cane P.A., Medley G.F., Nokes D.J. (2013). The Source of Respiratory Syncytial Virus Infection In Infants: A Household Cohort Study In Rural Kenya. J. Infect. Dis..

[16] Walker P.J., Siddell S.G., Lefkowitz E.J., Mushegian A.R., Adriaenssens E.M., Dempsey D.M., Dutilh B.E., Harrach B., Harrison R.L., Hendrickson R.C. (2020). Changes to Virus Taxonomy and the Statutes Ratified by the International Committee on Taxonomy of Viruses. Arch. Virol..

[17] Woo P.C.Y., Huang Y., Lau S.K.P., Yuen K.-Y. (2010). Coronavirus Genomics and Bioinformatics Analysis. Viruses.

[18] Lai M.M. (1992). RNA Recombination in Animal and Plant Viruses. Microbiol. Rev..

[19] Pasternak A., Spaan W.J.M., Snijder E. (2006). Nidovirus Transcription: How to make sense…?. J. Gen. Virol..

[20] Li X., Luk H.K.H., Lau S.K.P., Woo P.C.Y. (2019). Human Coronaviruses: General Features. Reference Module in Biomedical Sciences.

[21] Hulswit R.J.G., De Haan C.A.M., Bosch B.J. (2016). Coronavirus Spike Protein and Tropism Changes. Adv. Virus Res..

[22] De Haan C.A.M., Rottier P.J.M. (2005). Molecular Interactions in the Assembly of Coronaviruses. Adv. Virus Res..

[23] Xu R.H., He J.F., Evans M.R., Peng G.W., Field H.E., Yu D.W., Lee C.K., Luo H.M., Lin W.S., Lin P. (2004). Epidemiologic Clues to SARS Origin in China. Emerg. Infect. Dis..

[24] Woo P.C.Y., Lau S.K., Yuen K.-Y. (2006). Infectious Diseases Emerging from Chinese Wet-Markets: Zoonotic Origins of Severe Respiratory Viral Infections. Curr. Opin. Infect. Dis..

[25] Corman V.M., Muth D., Niemeyer D., Drosten C. (2018). Hosts and Sources of Endemic Human Coronaviruses. Adv. Virus Res..

[26] Ksiazek T.G., Erdman D., Goldsmith C.S., Zaki S.R., Peret T., Emery S., Tong S., Urbani C., Comer J.A., Lim W. (2003). A Novel Coronavirus Associated with Severe Acute Respiratory Syndrome. N. Engl. J. Med..

[27] Drosten C., Günther S., Preiser W., Van Der Werf S., Brodt H.-R., Becker S., Rabenau H., Panning M., Kolesnikova L., Fouchier R.A.M. (2003). Identification of a Novel Coronavirus in Patients with Severe Acute Respiratory Syndrome. N. Engl. J. Med..

[28] Peiris J.S.M., Guan Y., Yuen K.-Y. (2004). Severe Acute Respiratory Syndrome. Nat. Med..

[29] McIntosh K., Becker W.B., Chanock R.M. (1967). Growth in Suckling-Mouse Brain of “IBV-like” Viruses from Patients with Upper Respiratory Tract Disease. Proc. Natl. Acad. Sci. USA.

[30] Bradburne A.F., Bynoe M.L., Tyrrell D.A. (1967). Effects of a “New” Human Respiratory Virus in Volunteers. BMJ.

[31] Hamre D., Procknow J.J. (1966). A New Virus Isolated from the Human Respiratory Tract. Proc. Soc. Exp. Biol. Med..

[32] Van Der Hoek L., Pyrc K., Jebbink M.F., Vermeulen-Oost W., Berkhout R.J., Wolthers K.C., Wertheim-van Dillen P.M.E., Kaandorp J., Spaargaren J., Berkhout B. (2004). Identification of a New Human Coronavirus. Nat. Med..

[33] Woo P.C.Y., Lau S.K.P., Chu C., Chan K., Tsoi H., Huang Y., Wong B.H.L., Poon R.W.S., Cai J.J., Luk W. (2005). Characterization and Complete Genome Sequence of a Novel Coronavirus, Coronavirus HKU1, from Patients with Pneumonia. J. Virol..

[34] Wong S., Lau S., Woo P., Yuen K.-Y. (2007). Bats as a Continuing Source of Emerging Infections in Humans. Rev. Med. Virol..

[35] Woo P.C.Y., Lau S.K.P., Lam C.S.F., Lau C.C.Y., Tsang A.K.L., Lau J.H.N., Bai R., Teng J.L.L., Tsang C.C.C., Wang M. (2012). Discovery of Seven Novel Mammalian and Avian Coronaviruses in the Genus Deltacoronavirus Supports Bat Coronaviruses as the Gene Source of Alphacoronavirus and Betacoronavirus and Avian Coronaviruses as the Gene Source of Gammacoronavirus and Deltacoronavirus. J. Virol..

[36] Chan J.F.-W., To K.K.-W., Tse H., Jin D.Y., Yuen K.Y. (2013). Interspecies Transmission and Emergence of Novel Viruses: Lessons from Bats and Birds. Trends Microbiol..

[37] Vijgen L., Keyaerts E., Moës E., Thoelen I., Wollants E., Lemey P., Vandamme A.-M., Van Ranst M. (2005). Complete Genomic Sequence of Human Coronavirus OC43: Molecular Clock Analysis Suggests a Relatively Recent Zoonotic Coronavirus Transmission Event. J. Virol..

[38] Vijgen L., Keyaerts E., Lemey P., Maes P., Van Reeth K., Nauwynck H., Pensaert M., Van Ranst M. (2006). Evolutionary History of the Closely Related Group 2 Coronaviruses: Porcine Hemagglutinating Encephalomyelitis Virus, Bovine Coronavirus, and Human Coronavirus OC43. J. Virol..

[39] Corman V.M., Eckerle I., Memish Z.A., Liljander A.M., Dijkman R., Jonsdottir H.R., Ngeiywa K.J.Z.J., Kamau E., Younan M., Al Masri M. (2016). Link of a ubiquitous human coronavirus to dromedary camels. Proc. Natl. Acad. Sci. USA.

[40] Corman V.M., Baldwin H.J., Tateno A.F., Zerbinati R.M., Annan A., Owusu M., Nkrumah E.E., Maganga G.D., Oppong S., Adu-Sarkodie Y. (2015). Evidence for an Ancestral Association of Human Coronavirus 229E with Bats. J. Virol..

[41] Pfefferle S., Oppong S., Drexler J.F., Gloza-Rausch F., Ipsen A., Seebens A., Müller M.A., Annan A., Vallo P., Adu-Sarkodie Y. (2009). Distant Relatives of Severe Acute Respiratory Syndrome Coronavirus and Close Relatives of Human Coronavirus 229E in Bats, Ghana. Emerg. Infect. Dis..

[42] Forni D., Cagliani R., Clerici M., Sironi M. (2016). Molecular Evolution of Human Coronavirus Genomes. Trends Microbiol..

[43] Vakulenko Y., Deviatkin A., Lukashev A. (2019). The Effect of Sample Bias and Experimental Artefacts on the Statistical Phylogenetic Analysis of Picornaviruses. Viruses.

[44] Hall M.D., Woolhouse M., Rambaut A. (2016). The Effects of Sampling Strategy on the Quality of Reconstruction of Viral Population Dynamics Using Bayesian Skyline Family Coalescent Methods: A simulation study. Virus Evol..

[45] Chernomor O., Minh B.Q., Forest F., Klaere S., Ingram T., Henzinger M., Von Haeseler A. (2014). Split Diversity in Constrained Conservation Prioritization Using Integer Linear Programming. Methods Ecol. Evol..

[46] Woo P.C.Y., Lau S.K.P., Yip C.C.Y., Huang Y., Tsoi H.-W., Chan K.-H., Yuen K.-Y. (2006). Comparative Analysis of 22 Coronavirus HKU1 Genomes Reveals a Novel Genotype and Evidence of Natural Recombination in Coronavirus HKU1. J. Virol..

[47] Katoh K., Standley D.M. (2013). MAFFT Multiple Sequence Alignment Software Version 7: Improvements in Performance and Usability. Mol. Biol. Evol..

[48] Larsson A. (2014). AliView: A fast and lightweight alignment viewer and editor for large datasets. Bioinformatics.

[49] Minh B.Q., Schmidt H.A., Chernomor O., Schrempf D., Woodhams M.D., von Haeseler A., Lanfear R. (2020). IQ-TREE 2: New Models and Efficient Methods for Phylogenetic Inference in the Genomic Era. Mol. Biol. Evol..

[50] Kalyaanamoorthy S., Minh B.Q., Wong T.K.F., Von Haeseler A., Jermiin L.S. (2017). ModelFinder: Fast Model Selection for Accurate Phylogenetic Estimates. Nat. Methods.

[51] Hoang D.T., Chernomor O., Von Haeseler A., Minh B.Q., Vinh L.S. (2018). UFBoot2: Improving the Ultrafast Bootstrap Approximation. Mol. Biol. Evol..

[52] Rambaut A., Lam T.T., Max Carvalho L., Pybus O.G. (2016). Exploring the temporal structure of heterochronous sequences using TempEst (formerly Path-O-Gen). Virus Evol..

[53] Kumar S., Stecher G., Li M., Knyaz C., Tamura K. (2018). MEGA X: Molecular Evolutionary Genetics Analysis across Computing Platforms. Mol. Biol. Evol..

[54] Tamura K., Nei M., Kumar S. (2004). Prospects for Inferring Very Large Phylogenies by Using the Neighbor-Joining Method. Proc. Natl. Acad. Sci. USA.

[55] Lai M.M., Cavanagh D. (1997). The Molecular Biology of Coronaviruses. Adv. Virus Res..

[56] Graham R.L., Baric R.S. (2010). Recombination, Reservoirs, and the Modular Spike: Mechanisms of Coronavirus Cross-Species Transmission. J. Virol..

[57] Vakulenko Y., Deviatkin A., Drexler J., Lukashev A. (2021). Modular Evolution of Coronavirus Genomes. Viruses.

[58] Posada D., Crandall K.A. (2002). The Effect of Recombination on the Accuracy of Phylogeny Estimation. J. Mol. Evol..

[59] Didelot X., Maiden M.C. (2010). Impact of Recombination on Bacterial Evolution. Trends Microbiol..

[60] Jo W.K., Drosten C., Drexler J.F. (2021). The Evolutionary Dynamics of Endemic Human Coronaviruses. Virus Evol..

[61] Martin D.P., Murrell B., Golden M., Khoosal A., Muhire B. (2015). RDP4: Detection and Analysis of Recombination Patterns in Virus Genomes. Virus Evol..

[62] Suchard M.A., Lemey P., Baele G., Ayres D.L., Drummond A.J., Rambaut A. (2018). Bayesian Phylogenetic and Phylodynamic Data Integration Using BEAST 1.10. Virus Evol..

[63] Ayres D.L., Darling A., Zwickl D.J., Beerli P., Holder M., Lewis P.O., Huelsenbeck J.P., Ronquist F., Swofford D.L., Cummings M.P. (2011). BEAGLE: An Application Programming Interface and High-Performance Computing Library for Statistical Phylogenetics. Syst. Biol..

[64] Lemey P., Rambaut A., Drummond A.J., Suchard M.A. (2009). Bayesian Phylogeography Finds Its Roots. PLoS Comput. Biol..

[65] Shapiro B., Rambaut A., Drummond A. (2005). Choosing Appropriate Substitution Models for the Phylogenetic Analysis of Protein-Coding Sequences. Mol. Biol. Evol..

[66] Drummond A.J., Rambaut A., Shapiro B., Pybus O.G. (2005). Bayesian Coalescent Inference of Past Population Dynamics from Molecular Sequences. Mol. Biol. Evol..

[67] Drummond A.J., Ho S.Y.W., Phillips M.J., Rambaut A. (2006). Relaxed Phylogenetics and Dating with Confidence. PLoS Biol..

[68] Rambaut A., Drummond A.J., Xie D., Baele G., Suchard M.A. (2018). Posterior Summarization in Bayesian Phylogenetics Using Tracer 1.7. Syst. Biol..

[69] Yu G., Smith D.K., Zhu H., Guan Y., Lam T.T.Y. (2017). Ggtree: An R Package for Visualization and Annotation of Phylogenetic Trees with Their Covariates and Other Associated Data. Methods Ecol. Evol..

[70] Minin V.N., Suchard M.A. (2007). Counting Labeled Transitions in Continuous-Time Markov Models of Evolution. J. Math. Biol..

[71] Smith M.D., Wertheim J.O., Weaver S., Murrell B., Scheffler K., Pond S.L.K. (2015). Less Is More: An Adaptive Branch-Site Random Effects Model for Efficient Detection of Episodic Diversifying Selection. Mol. Biol. Evol..

[72] Murrell B., Weaver S., Smith M.D., Wertheim J.O., Murrell S., Aylward A., Eren K., Pollner T., Martin D.P., Smith D.M. (2015). Gene-Wide Identification of Episodic Selection. Mol. Biol. Evol..

[73] Trovão N.S., Khan S.M., Lemey P., Nelson M.I., Cherry J.L. (2022). Evolution of Influenza A Virus Hemagglutinin H1 and H3 across Host Species. bioRxiv.

[74] Tagliamonte M.S., Abid N., Borocci S., SanGiovanni E., Ostrov D.A., Pond S.L.K., Salemi M., Chillemi G., Mavian C. (2020). Multiple Recombination Events and Strong Purifying Selection at the Origin of SARS-CoV-2 Spike Glycoprotein Increased Correlated Dynamic Movements. Int. J. Mol. Sci..

[75] Lau S.K.P., Lee P., Tsang A.K.L., Yip C.C.Y., Tse H., Lee R.A., So L.-Y., Lau Y.-L., Chan K.-H., Woo P.C.Y. (2011). Molecular Epidemiology of Human Coronavirus OC43 Reveals Evolution of Different Genotypes over Time and Recent Emergence of a Novel Genotype due to Natural Recombination. J. Virol..

[76] Pyrc K., Dijkman R., Deng L., Jebbink M.F., Ross H.A., Berkhout B., van der Hoek L. (2006). Mosaic Structure of Human Coronavirus NL63, One Thousand Years of Evolution. J. Mol. Biol..

[77] Klinakis A., Cournia Z., Rampias T. (2021). N-Terminal Domain Mutations of the Spike Protein Are Structurally Implicated in Epitope Recognition in Emerging SARS-CoV-2 Strains. Comput. Struct. Biotechnol. J..

[78] Cui Z., Liu P., Wang N., Wang L., Fan K., Zhu Q., Wang K., Chen R., Feng R., Jia Z. (2022). Structural and Functional Characterizations of Infectivity and Immune Evasion of SARS-CoV-2 Omicron. Cell.

[79] Cao Y., Wang J., Jian F., Xiao T., Song W., Yisimayi A., Huang W., Li Q., Wang P., An R. (2021). Omicron Escapes the Majority of Existing SARS-CoV-2 Neutralizing Antibodies. Nature.

[80] Kumar S., Thambiraja T.S., Karuppanan K., Subramaniam G. (2021). Omicron and Delta Variant of SARS-CoV-2: A Comparative Computational Study of Spike Protein. J. Med. Virol..

[81] Maaroufi H. (2022). The N764K and N856K Mutations in SARS-CoV-2 Omicron S Protein Generate Potential Cleavage Sites for SKI-1/S1P Protease. bioRxiv.

[82] Dudas G., Carvalho L.M., Rambaut A., Bedford T. (2018). MERS-CoV Spillover at the Camel-Human Interface. eLife.

[83] VanInsberghe D., Neish A.S., Lowen A.C., Koelle K. (2021). Recombinant SARS-CoV-2 Genomes Are Currently Circulating at Low Levels. bioRxiv Prepr. Serv. Biol..

[84] Simon-Loriere E., Holmes E.C. (2011). Why Do RNA Viruses Recombine?. Nat. Rev. Genet..

[85] Duffy S. (2018). Why Are RNA Virus Mutation Rates so Damn High?. PLoS Biol..

[86] Kistler K.E., Bedford T. (2021). Evidence for Adaptive Evolution in the Receptor-Binding Domain of Seasonal Coronaviruses OC43 and 229e. eLife.

[87] Müller N.F., Kistler K.E., Bedford T. (2021). Recombination Patterns in Coronaviruses. bioRxiv Prepr. Serv. Biol..

[88] Al-Khannaq M.N., Ng K.T., Oong X.Y., Pang Y.K., Takebe Y., Chook J.B., Hanafi N.S., Kamarulzaman A., Tee K.K. (2016). Molecular Epidemiology and Evolutionary Histories of Human Coronavirus OC43 and HKU1 among Patients with Upper Respiratory Tract Infections in Kuala Lumpur, Malaysia. Virol. J..

[89] Marin J., Hedges S.B. (2018). Undersampling Genomes Has Biased Time and Rate Estimates Throughout the Tree of Life. Mol. Biol. Evol..

[90] Flint J., Racaniello V.R., Skalka A.M., Rall G. (2015). Principles of Virology, Bundle. Princ. Virol. Bundle.

[91] Banner L.R., Mc Lai M. (1991). Random Nature of Coronavirus RNA Recombination in the Absence of Selection Pressure. Virology.

