# Supplementary figures and images for: Origins and Evolution of Seasonal Human Coronaviruses
Source: Viruses. 2022 Jul 15;14(7):1551. doi: 10.3390/v14071551 (PMC9320361; doi:10.3390/v14071551)

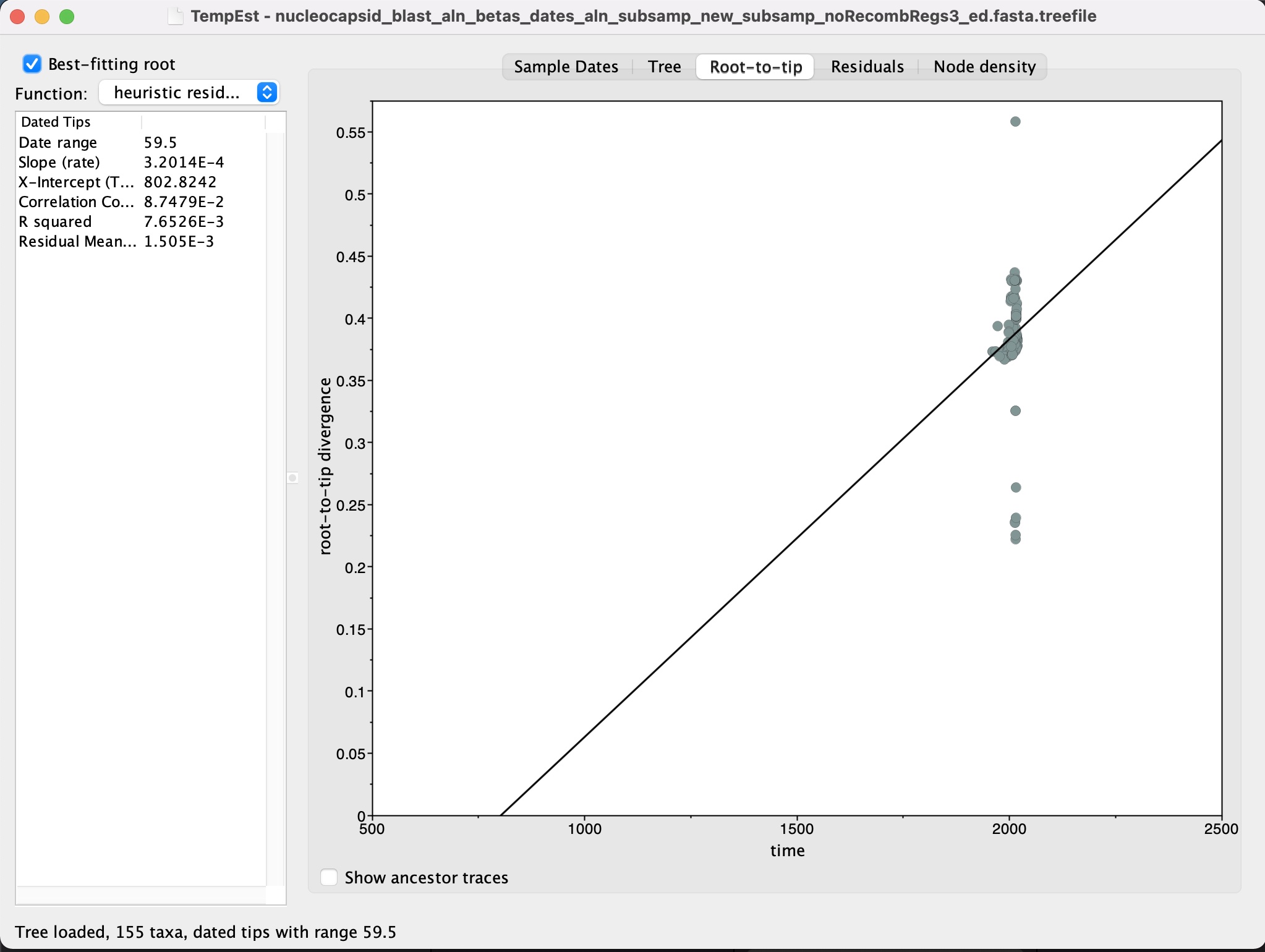

Supplement: Supplementary file 1 [file viruses-14-01551-s001.zip › TempEst/nucleo_beta.jpg]

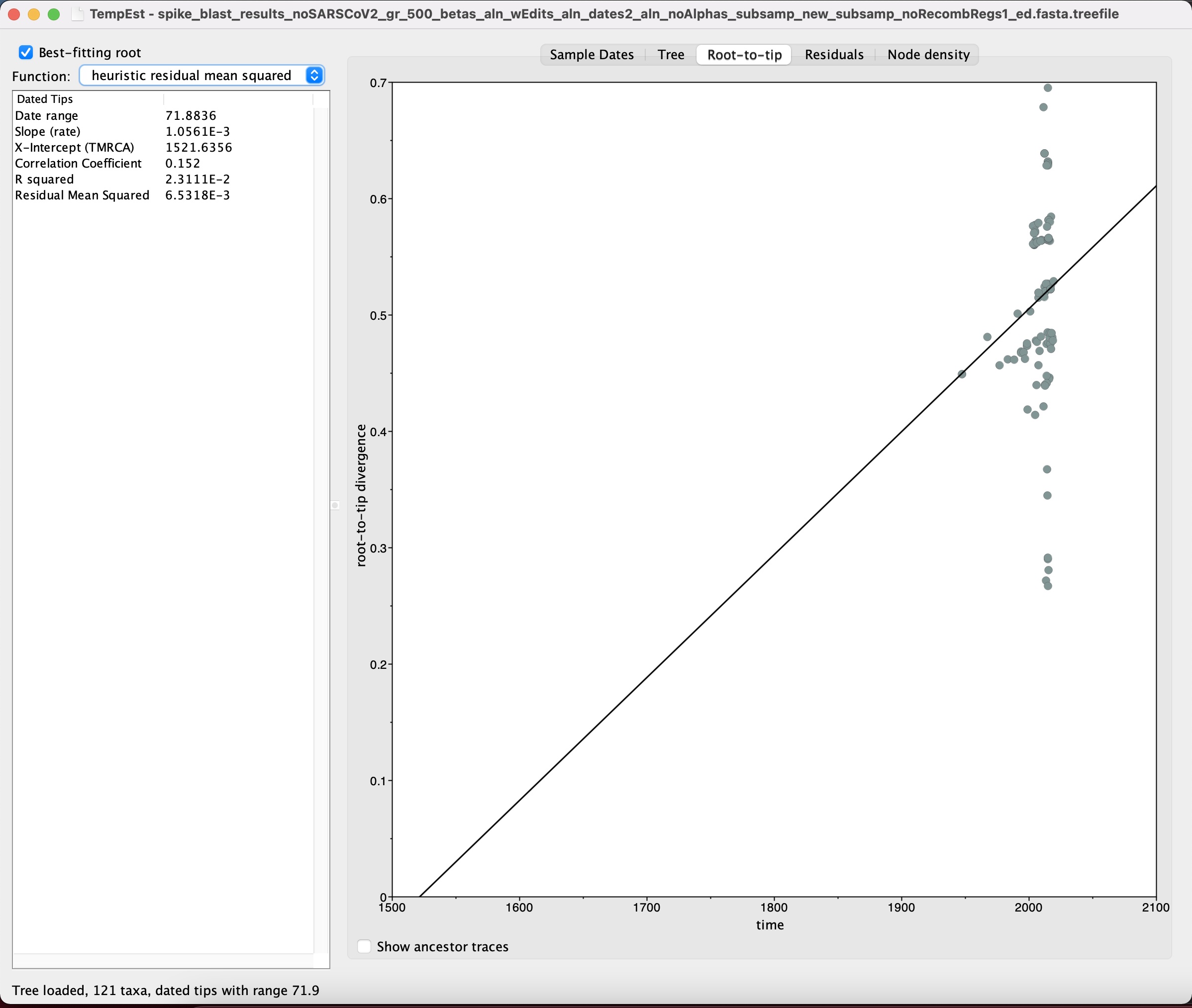

Supplement: Supplementary file 1 [file viruses-14-01551-s001.zip › TempEst/spike_beta.jpg]

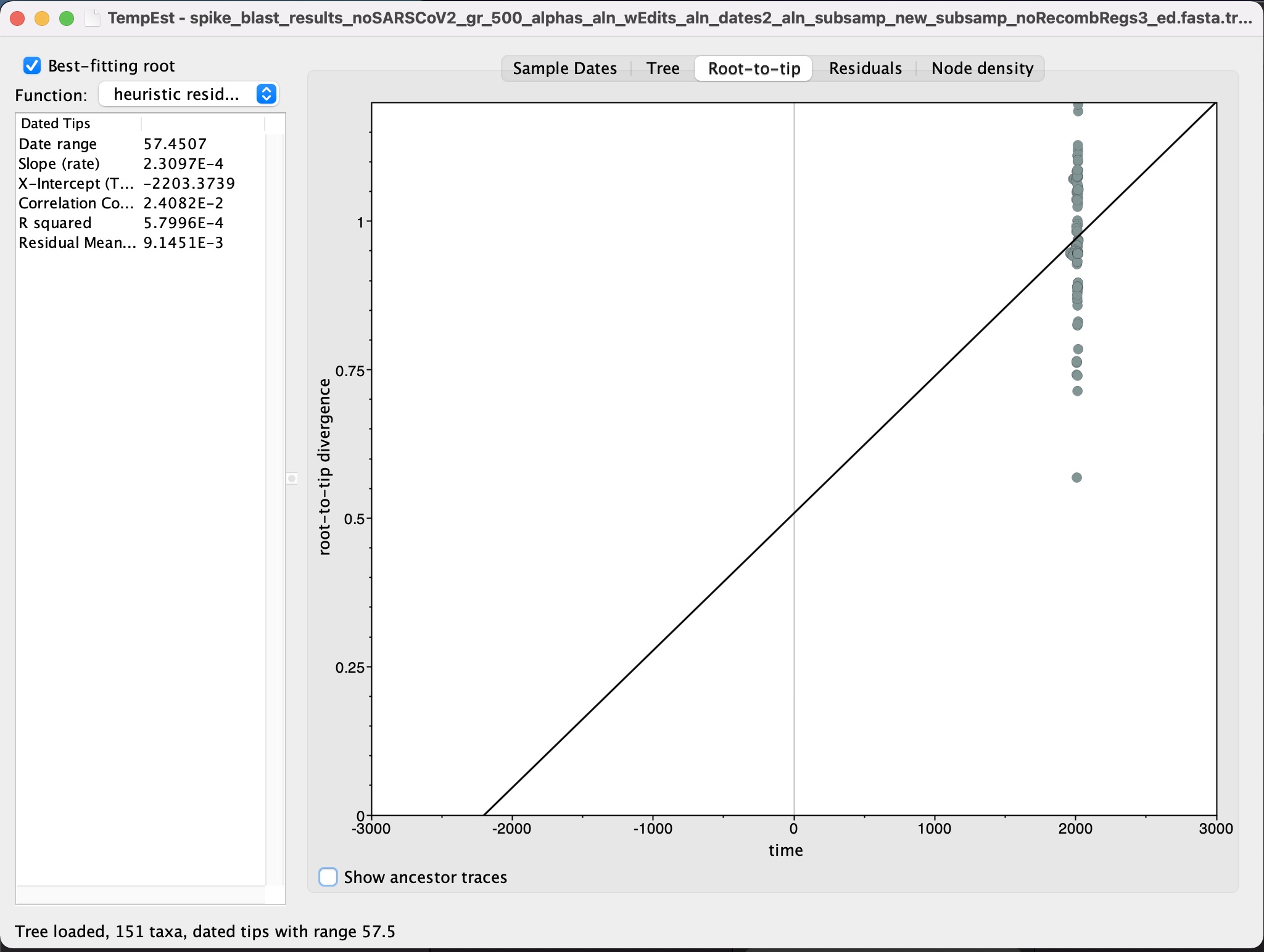

Supplement: Supplementary file 1 [file viruses-14-01551-s001.zip › TempEst/spike_alpha.jpg]

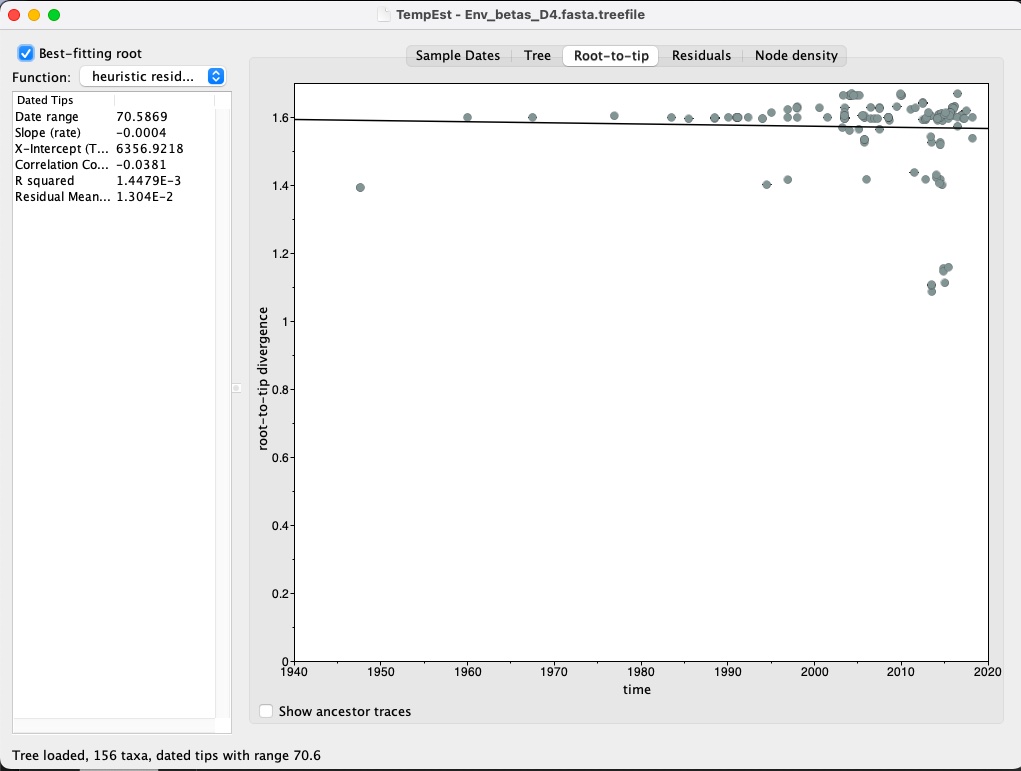

Supplement: Supplementary file 1 [file viruses-14-01551-s001.zip › TempEst/env_beta.jpg]

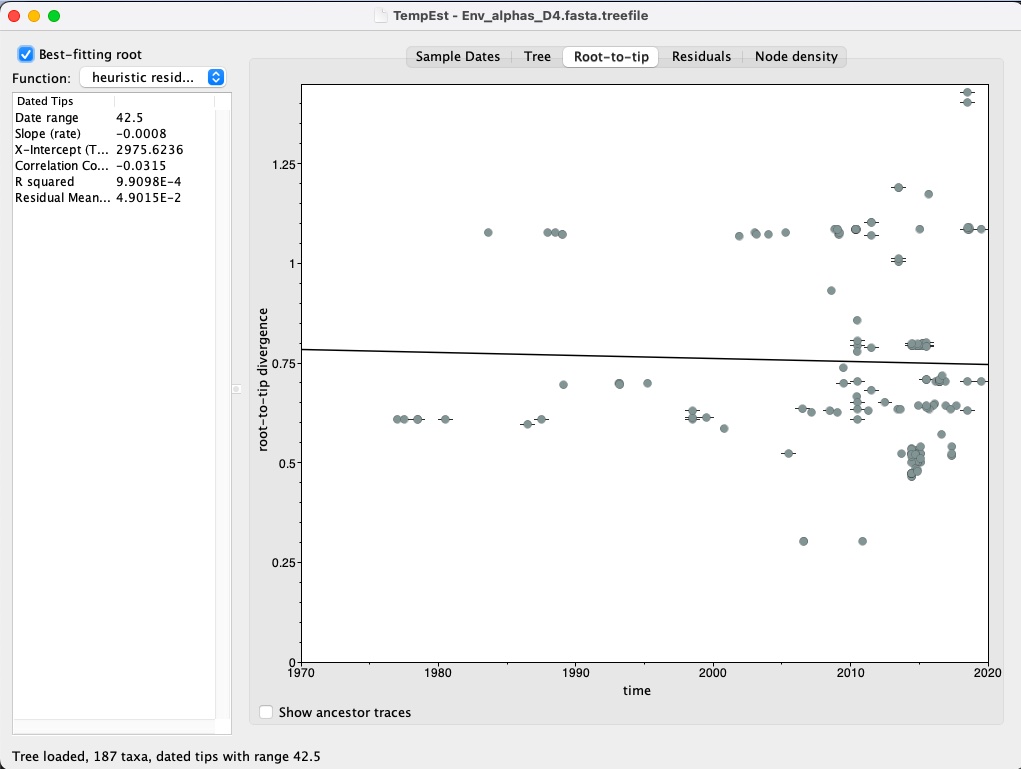

Supplement: Supplementary file 1 [file viruses-14-01551-s001.zip › TempEst/env_alpha.jpg]

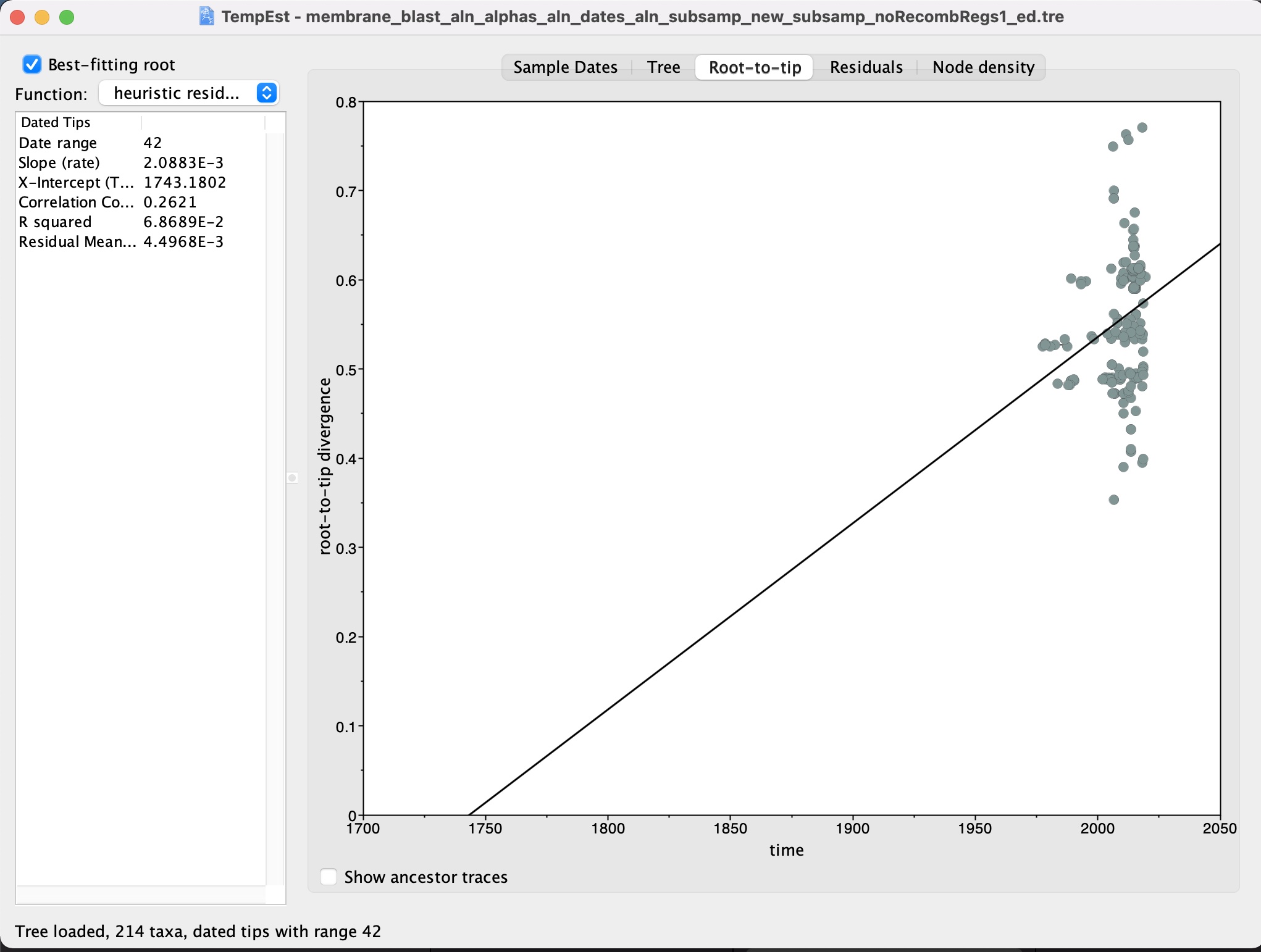

Supplement: Supplementary file 1 [file viruses-14-01551-s001.zip › TempEst/memb_alpha.jpg]

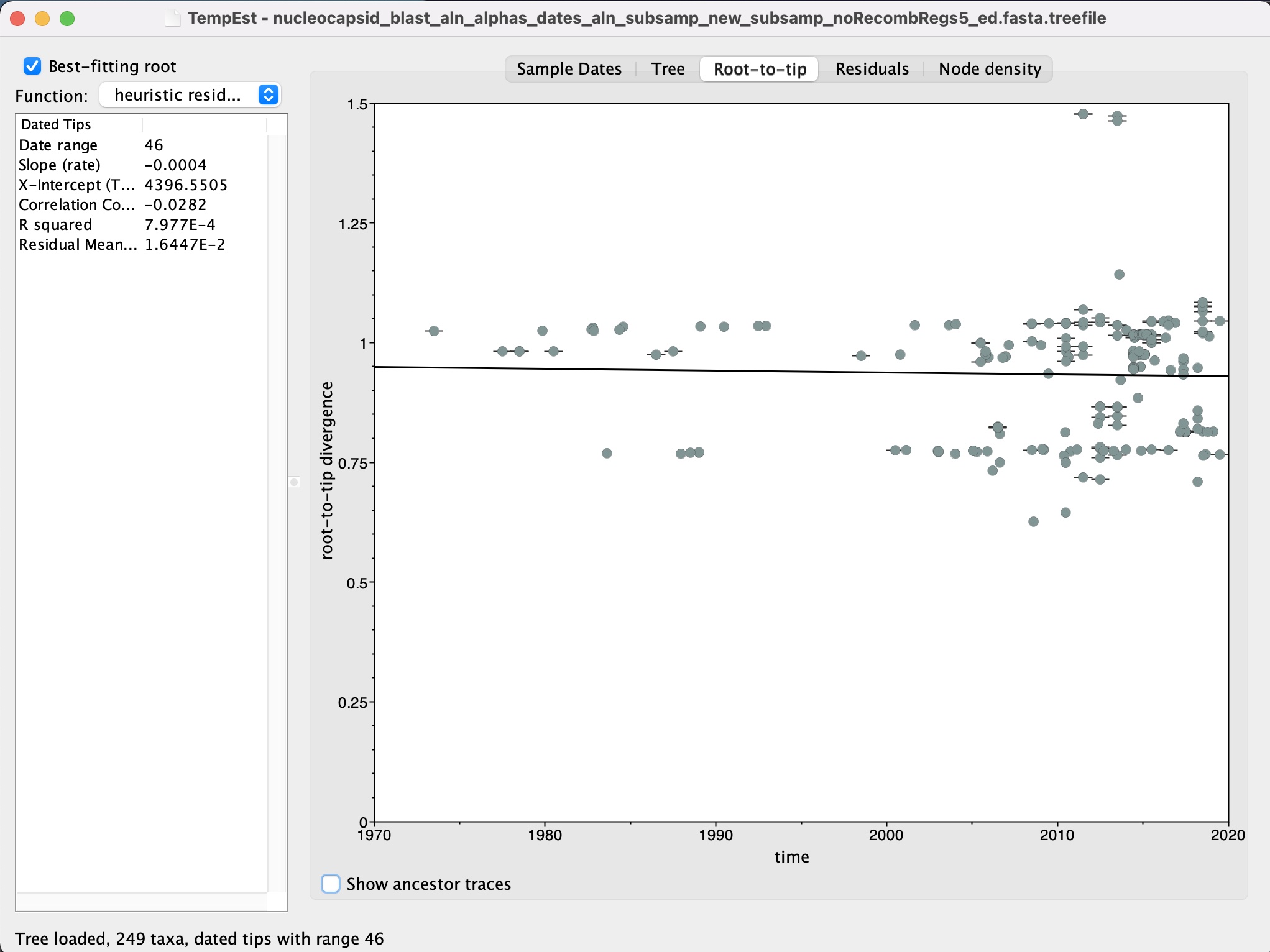

Supplement: Supplementary file 1 [file viruses-14-01551-s001.zip › TempEst/nucleo_alpha.jpg]

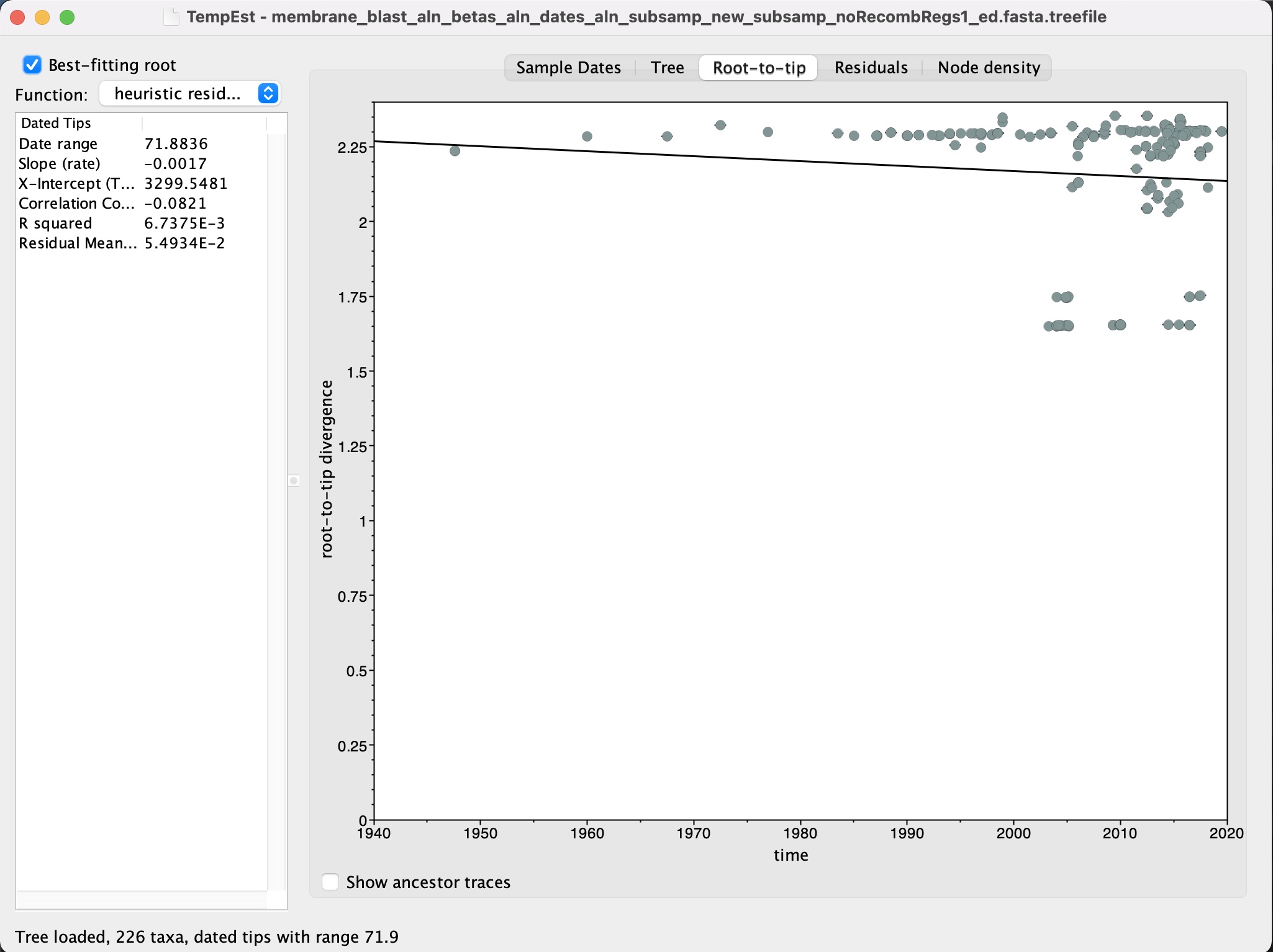

Supplement: Supplementary file 1 [file viruses-14-01551-s001.zip › TempEst/memb_beta.jpg]

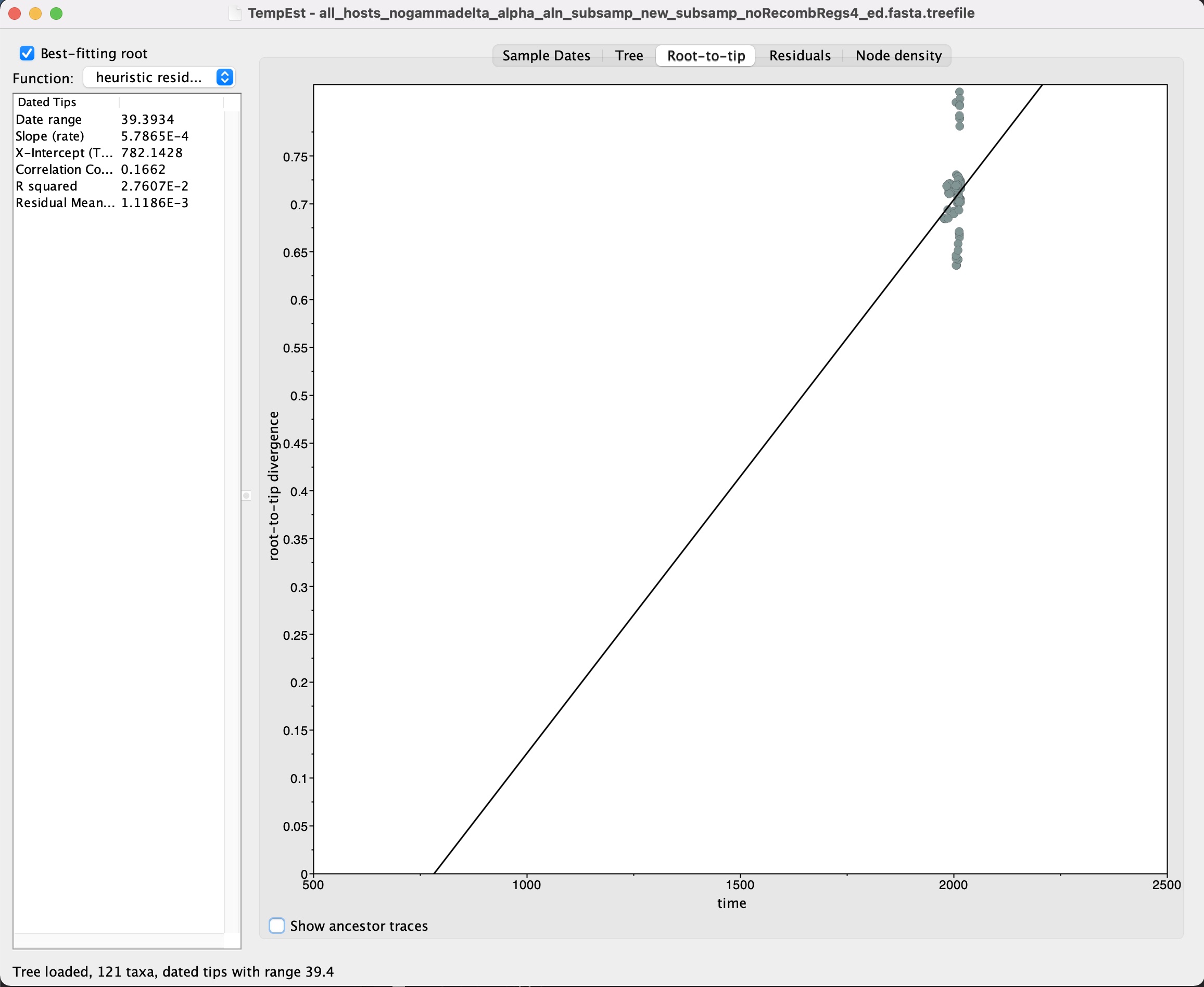

Supplement: Supplementary file 1 [file viruses-14-01551-s001.zip › TempEst/wgs_alpha.jpg]

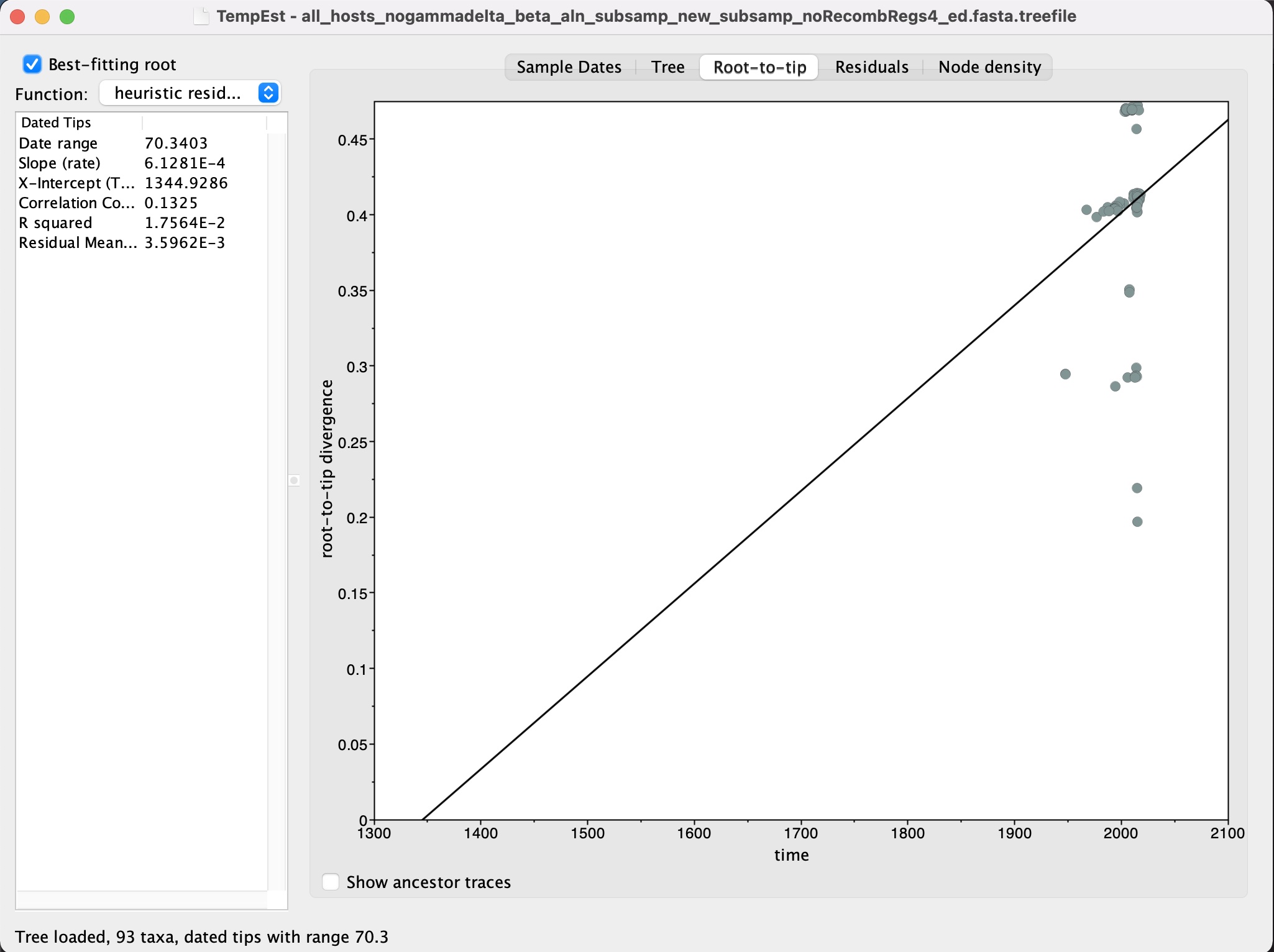

Supplement: Supplementary file 1 [file viruses-14-01551-s001.zip › TempEst/wgs_beta.jpg]
